# Social movements and collective behavior: an integration of meta-analysis and systematic review of social psychology studies

**DOI:** 10.3389/fpsyg.2023.1096877

**Published:** 2023-04-21

**Authors:** Silvia da Costa, Dario Páez, Mariacarla Martí-González, Virginia Díaz, Pierre Bouchat

**Affiliations:** ^1^Faculty of Social and Human Sciences, Department of Psychology and Sociology, University of Zaragoza, Teruel, Spain; ^2^Faculty of Psychology, Department of Social Psychology, University of Basque Country, San Sebastian, Spain; ^3^Faculty of Education and Social Sciences, Andrés Bello University, Santiago, Chile; ^4^Department of Psychology and Sociology, University of Zaragoza, Zaragoza, Spain; ^5^Department of Education, University of Cantabria, Santander, Spain; ^6^Laboratoire Perseus, University of Lorraine, Nancy, France

**Keywords:** collective behaviors, collective gatherings, meta-analysis, systematic review, social psychology, social movements

## Abstract

**Background:**

The impact of social movements (SMs) and collective behavior (CB) supports the relevance of approaching this phenomenon from social psychology. Several systematic reviews (10) and meta-analyses (6) have been carried out in the 21st century, but there is a lack of integration.

**Aim:**

This study seeks to review the patterns of CB and corroborate the psychosocial factors that explain participation in CB and SMs, as well as the long-term psychological effects of participating in them.

**Method:**

A systematic search was carried out in the databases Web of Science, Scopus, ProQuest, ScienceDirect, Willey Online Library, EBSCO, and JSTOR for articles dated between 1969 and 2022. We searched for meta-analyses and systematic reviews that empirically evaluated social movements and collective behavior. Of the 494 initial records, after scanning and eligibility phases, 16 meta-analyses and systematic reviews were analyzed in the present work.

**Results:**

The evidence reviewed shows that participation in collective gatherings and CB are common. A cross-cultural survey suggests that collective gatherings are mostly of a leisure type, to a lesser extent religious and sporting, and to an even lesser extent, demonstrations and large religious rites. World Value surveys found that one to three persons out of 10 participate in protests or CB related to SMs and four out of 10 movements achieved some kind of success. Studies challenged that CBs were characterized by unanimity of beliefs, identification and behavior, generalized excitement, as well as mass panic and riot after catastrophes. Only two out of 10 CB are violent. Meta-analysis and systematic reviews confirm that participation in CB and SMs was associated with (a) intergroup conflict and realistic threat (*r* = 0.30); (b) positive attitudes, expectations, or agreement with goals or collective motive (*r* = 0.44); (c) cognitive fraternal relative deprivation (*r* = 0.25); (d) collective efficacy (*r* = 0.36); (e) collective identity (*r* = 0.34); (f) emotions and affective relative deprivation (*r* = 0.35); (g) moral conviction and threat to moral (*r* = 0.29); and (h) disagreement with system justification belief (*r* = −0.26). Participation in successful CB and SMs provokes positive changes in emotions, social identity and social relationships, values and beliefs, and empowerment, as well as negative effects such as depression, stress, burnout, and disempowerment related to the failures of SMs.

**Conclusion:**

Studies confirm the importance of explanatory factors for SMs, with data from various cultural regions. There is a lack of systematic studies of CB as well as meta-analyses and more culturally diverse studies of the effects of participation in them.

## 1. Introduction

This study examines the conceptualizations of collective behaviors (CB) and social movements (SMs). The study aims to provide an integrative vision of all types of CB, not only those related to social protest movements. On the one hand, demonstrations and collective gatherings linked to festivities, sports, and other “non-political” events share behavioral and psychological patterns with protest demonstrations. On the other hand, ludic events such as carnivals or mass sports easily become channels of expression of social protest. Classical studies such as those of Bakhtin, and also contemporary studies, show how carnivals serve as a form of social protest (see, for example, the carnivals in Uruguay, Remedi, 2020). The text is organized following the main explanatory theories of SMs based on the macrosocial organizing principles of social movements (van Zomeren, 2016), such as identity, opposition, and totality (Touraine, 1978), and the resources and opportunities for collective action (Tarrow, 1997). It also draws on the more micro-social and psychological theories of CB and SMs, such as the role of expectations, expected value, and rational logic of collective action (Klandermans, 1984; Gamson, 1992). It is also based on the Social Identity Model of Collective Action (van Zomeren et al., 2008). This model includes, as motivational explanatory variables of SMs, social and collective identities (Tajfel and Turner, 1986; Drury and Reicher, 2020, collective relative deprivation and efficacy (Vestergren et al., 2017), and positive emotions such as hope and negative ones such as moral anger (Jasper, 2011) and moral obligation (Sabucedo et al., 2018). Finally, ideological factors of CBs and SMs are taken into account, such as system justification beliefs (Jost et al., 2017) and collective action frames (Benford and Snow, 2000). From a psychosocial articulation perspective, structural factors determine psychological processes (motivational, affective, and thinking), which in turn produce group phenomena, such as CB—in a retroactive loop. Finally, the short-term effects of CB as well as medium- and long-term psychological effects of participation in SMs will be examined.

### 1.1. Conceptualization of social movements and collective behaviors

Before and during the COVID-19 pandemic, collective outbursts of protest and large mobilizations have shaken Chile, Colombia, and other countries. In recent decades, CB of protests and SMs have occurred in more than 180 countries, including 99% of the world's population. It is estimated that nearly 5,000 revolts that have occurred in 158 countries during the COVID-19 have demanded recognition of economic and social rights, in addition to showing the underlying vulnerability and social inequities with a negative economic impact of losses of 15 billion dollars (Ortiz et al., 2022). According to Tarrow (1997), SMs are collective challenges based on common goals and on social solidarity, which both occur in sustained interaction with elites, opponents, and authorities. Long-lasting mass movements emerge as a collective response to the discomfort created by a social problem or around a social conflict. SMs can be defined as a collective or group in which there is interaction—largely informal—that acts with some continuity to promote or resist change in the society of which it is a part. Acting with continuity implies a certain degree of organization, while at the same time, it makes possible prolongation of group identity, allowing the development of shared beliefs and values, i.e., an ideology. The development of a sense of unity or collective identity enables common action or social mobilization both institutionally and extra-institutionally (Touraine, 1978; Tremblay et al., 2017).

The SMs or mass movements are built accordingly to the principle of identity (a), the principle of opposition (b), and the principle of totality (c). The first (a) refers to the people who define themselves as participants in it, the second (b) explains what the movement is fighting against, and the last (c) refers to the worldview or the objective it is trying to impose (Touraine, 1978). In addition, SMs: (1) rely on a strong organizational base—involving leaders, members, or followers, and formal or informal organizations and coalitions—to build and organize the movement; (2) pursue a political agenda or “common cause”; (3) engage in collective actions oriented toward clear objectives and use a variety of strategies to achieve their goals; (4) use interpretive frameworks to define a problematic situation in need of change to articulate a solution and raise awareness or motivate others to act or manifest their support; (5) develop themselves in relation to specific opportunities and follow a long life cycle that maintains some continuity over time; (6) take advantages of the tangible and intangible resources of individuals and groups; and (7) seek political, social, or cultural change (Touraine, 1978; Tremblay et al., 2017; Páez and da Costa, 2022). A classic example of this is the labor movement, which developed extra-institutional actions or revolts to bring about legal and political changes (right to unionize, 8-h day, limitation of child labor, and others). In the 19th century in Great Britain and the 20th century in Brazil, the labor movement developed itself not only in SMs of trade unionism but also became a party with political issues, the Labor party and the Working People's Party, respectively. Thus, urban movements of students, feminists, and ecologists, as well as those of political, religious, and ideological orientation are the most frequent ones (Páez and da Costa, 2022).

Collective behavior of protests can be considered as belonging to the action repertoire of SMs. In this way, participation in demonstrations, protests, meetings, or collective mass gatherings make up the field of CB. In turn, demonstrations or protests are forms of collective action linked to SMs and are defined as any temporary occupation by several people in an open space, public or private, which directly or indirectly includes the expression of political opinions (Filleule and Tartakowski, 2013). Thus, in collective gatherings, people are oriented toward an object of attention and possess some shared belief or objective. At the same time, the action is directed by formalized norms prescribed by the culture, although it can also arise as an emergent norm (Páez and da Costa, 2022). Some examples of these collective gatherings are the May 1st demonstration, the National holidays of different countries, concerts, and festivals. Durkheim (1912/2008) was one of the classical authors who studied this social arena (Pizarro et al., 2022 in this monograph).

According to McPhail and Wohlstein (1983, p. 581): Gatherings are not synonymous with collective behavior but provide circumstances in which it may occur. People in gatherings engage in a variety of individual behaviors and may also, occasionally, engage in what we term collective behavior—i.e., two or more persons engaged in one or more behaviors (e.g., orientation, locomotion, gesticulation, tactile manipulation, and/or vocalization) that can be judged common or convergent on one or more dimensions (e.g., direction, velocity, tempo, and/or substantive content).

In short, CB is defined as those behaviors determined by a person's membership in a social group and carried out together with members of that group.^1^ They have been characterized by their ephemeral, extra-institutional, and emergent (spontaneous, unplanned) nature. However, there is a continuity between conventional and extra-institutional forms of ceremonies and political protests—a rally or a legal protest can deviate into demonstrations that question institutions, as, for example, the demonstration that occurred in Washington that led to the occupation of the parliament (Páez and da Costa, 2022). We have taken into account that CB can also be developed in scenarios such as crowd and audience. The people who make up the crowd are together—face-to-face and acting spontaneously—sharing some object of attention or common purpose, such as expressing a workers' protest in front of a public building. LeBon (1986) was one of the classic authors who studied this theme. Finally, a collective of people who attend to a common object but who do not have to be in immediate physical proximity constitutes a public or audience. Media audiences and social networks for the public can be considered prototypical examples of mass behavior. Likewise, Tarde (2011) was one of the authors who studied the psychology of the masses as audiences.

If the collective gatherings are protest rallies, it is a demonstration generally framed within SMs. Similarly, if the collective gathering is a party or celebration, it is a parade or expressive collective ritual (e.g., the May Day or Gay Pride parade or Carnivals). However, these expressive collective rituals, such as Carnivals, can also express SMs of protest (Bennett et al., 2015; Han, 2022; Hernandez Burgos and Rina Simón, 2022; Páez and da Costa, 2022). Riots are collective gatherings involving crowds that commit individual or collective violence against people or property. Crowds can become demonstrations and demonstrations can become riots (McPhail and Wohlstein, 1983). In addition, hooliganism by sports fans is close to rioting. This is because hooligans not only use violence in their attempts to humiliate competing gangs supporting other club teams but also to draw attention to their social background and express grievances related to their social position (Drury, 2020). In addition, audiences can be remote participants of collective gatherings (for example, they participate vicariously in a funeral mourning such as that of Queen Elizabeth II of England or “Lady Di”), reacting spontaneously through social networks and generating a virtual crowd, as well as convening mass demonstrations or collective gatherings from them (Castells, 2012). Thus, SMs based on digital technologies are CB of audiences (Akfirat et al., 2021).

The impact of mass movements on social change is important and often positive too. General suffrage, including women's suffrage, the end of racist regimes, laws, and practices, as well as addressing climate change are all changes that are products of large SMs. A review of protests around the world during the 21st century found that more than four out of 10 movements achieved some kind of success, mostly on political, legal, and social rights issues, and to a lesser extent economic ones (Ortiz et al., 2022). We should bear in mind that six out of 10 protests are unsuccessful and that five to six out of 10 people refuse to participate in collective actions. In agreement, the World Values Survey studies, conducted during the years 2000, 2010, and 2017–2022, show that a minority mobilizes: to the question “have you participated in a peaceful demonstration last year”, between 12.5 and 15% respond affirmatively, in an illegal strike from 19 to 6%, have occupied buildings or factories 13%, and participated in boycotts 9%. About 30% agree that they could carry out these actions, but those who refuse to do so are between 57 and 63%.^2^

Social psychology literature has proposed different psychosocial explanatory factors for participation in CB and SMs, such as fraternal relative deprivation, socio-political opportunities and resources for mobilization, rational decisions, attitudes and expectations, collective efficacy, collective identity, frames of mobilization, emotions related to mobilization, moral conviction and threat to moral, and disagreement with system justification beliefs (Páez and da Costa, 2022). It has also been proposed that participation has medium- and long-term effects like positive changes in emotions, social identity and social relationships, values and beliefs, and empowerment, as well as negative effects such as depression, stress, burnout, and disempowerment related to failures (Vestergren et al., 2017; see Pizarro et al., 2022 in this monograph).

This study examines meta-analyses, systematic reviews, and specific studies that exist to date on the main variables and their correlates with the objectives of (1) reviewing common patterns of behavior, emotion, and cognition during CB, (2) psychosocial factors that explain participation in SMs and CB, and (3) the medium- and long-term psychological effects of participation in them.

**Figure 1:**
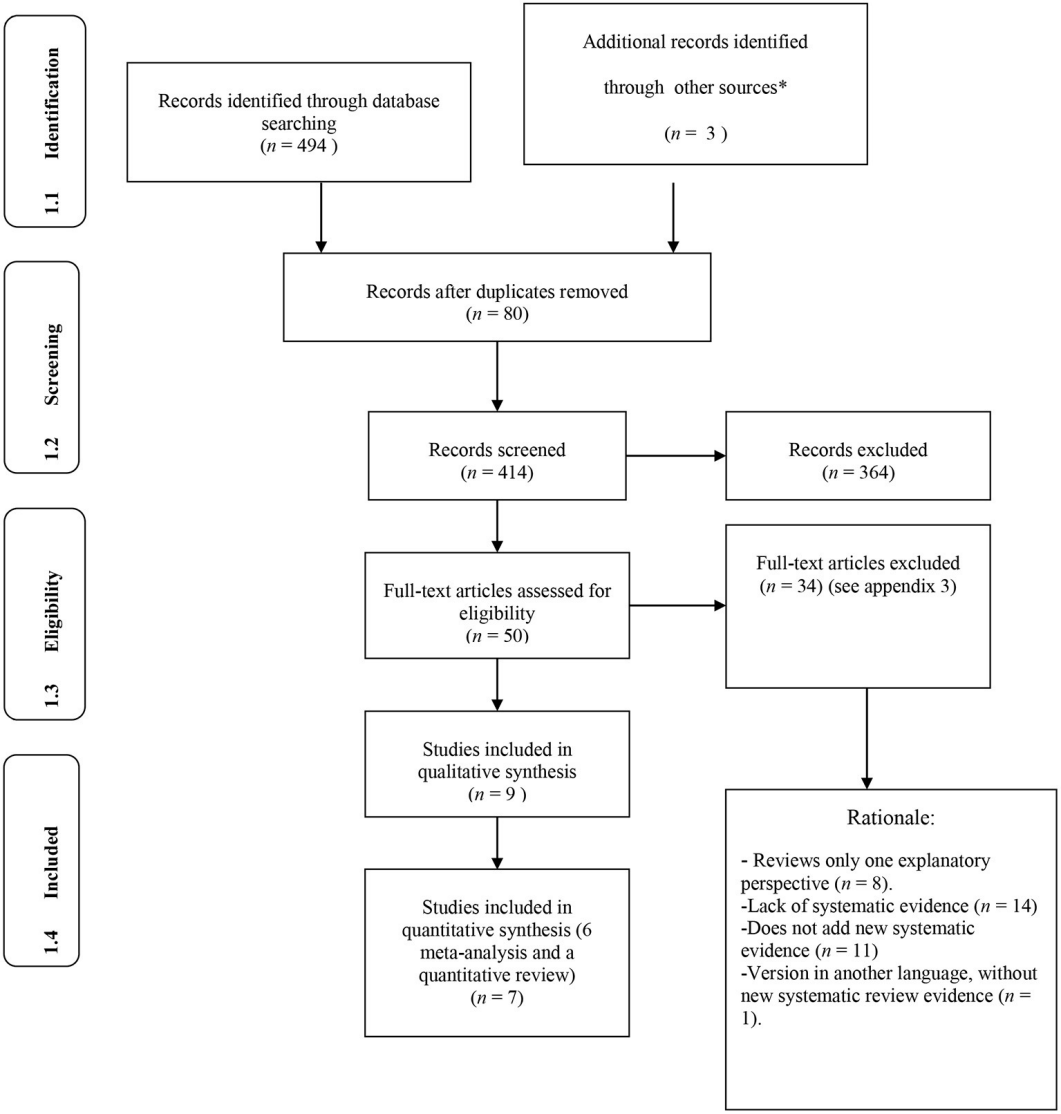
Systematic review PRISMA. *Blogs and personal communication.

This is, to our knowledge, the first text that integrates the classical observational literature on collective behavior, with the studies of social movements. In addition, also in a novel way to our understanding, studies on the collective behavior of all kinds are combined, not limited to the manifestations of political protests. It is also the first article to carry out an integration of meta-analysis, estimating a weighted average effect size for each psychosocial factor of the SMs, generally based on three pooled databases. A synthesis of systematic reviews is combined with all existing meta-analyses on factors linked to social mobilization showing a global panorama of these issues—as was done in the classic text by Milgram and Toch (1969)—escaping from the highly focused and limited approach of much of contemporary social psychology. Also noteworthy is the presentation of a glossary of terms (Appendix 1), of a protocol that provides operationalization of predictor variables, the frequency of participation in CB and SMs and outcomes (Appendix 2), and the provision of documents excluded in this article (see Figure 1 and Appendix 3).

## 2. Method

### 2.1. Procedure

#### 2.1.1. Protocol and registry

A protocol was drafted for this *a priori* review and inclusion criteria were developed before starting the search. For this, the guidelines and recommendations of PRISMA-P (Moher et al., 2015) were followed. The guiding questions for the systematic review have been: 1. Which are the factors that explain participation in CB and SMs and 2. Which are the psychological effects of participation in them?

##### 2.1.1.1. Eligibility criteria

Meta-analyses and systematic and specific reviews were included that described (a) studies on CB and SMs; (b) there was no limitation of age or type of CB or SMs; and (c) works published in Spanish, English, French, Portuguese, and Italian.

Concerning the exclusion criteria, the following were excluded: (a) those that were individual or single-case studies; (b) documents that were not peer-reviewed publications, including doctoral theses or gray literature; (c) those written in languages other than the five designated for selection; and (d) documents for which the full text was not available, nor the corresponding databases, nor through professional networks of academic personnel (e.g., “Google Scholar,” “ResearchGate,” or “Academia Edu”).

##### 2.1.1.2. Data sources and search

For the systematic search of the scientific literature, the following databases were consulted: Web of Science, Scopus, ProQuest, Science Direct, Willey Online Library, EBSCO, and JSTOR. The key words “Collective Behavior,” “Social Psychology,” “Social Movements,” “Meta-Analysis,” and “Systematic Review” (as well as their equivalents in the search languages) were used in the search strategies. Those terms were searched in combination using the Boolean connector “AND” and “OR”, specifying field category “Social Psychology” AND “Social Movements” AND “Meta-Analysis” OR “Systematic Review”; “Social Psychology” AND “Collective Behavior” AND “Meta-Analysis” OR “Systematic Review”, and with temporal limitation of publication. Terms such as collective action and political action were not included because we wanted to maintain the historical tradition of referring to collective behaviors and social movements—as defined in the classic chapter by Milgram and Toch in the Handbook of social psychology of 1969. The search years were from 1969, the last year in which a chapter on CB and SMs appeared in the Handbook of Social Psychology (Milgram and Toch, 1969, in Lindzey and Aronson, 1969), until 2022. The search took place in two periods of time, the first between January and May 2022 (Páez and da Costa, 2022) and the second between July and October 2022, by the authors of this article. Identified records to which some of the persons in the article had access, through academic contact, were added manually.

##### 2.1.1.3. Study selection and data extraction

The articles used in the review were selected from the PRISMA-P Search Diagram (Moher et al., 2015). Figure 1 shows the process of study selection. In the initial search, after applying the search strategy, 494 potential articles or chapters were found. Of those, 80 duplicate articles were eliminated and of the remaining 414, all those that were systematic reviews or meta-analyses (*n* = 50) were selected for an assessment of their eligibility by searching for information in the full text. Those that did not meet the inclusion criteria were discarded (*n* = 34), resulting in a total of 16 documents that met the requirements to be included in the systematic review.

##### 2.1.1.4. Quality assessment

Once it was ascertained that the articles examined the patterns of CB, motivational factors for participation in SMs and CB, and the psychological effects of participation in them, the full content of the articles was analyzed to see to what extent they met the quality criteria. The articles finally selected were published in peer-reviewed journals or chapters in books recognized for their quality (Handbook, Annual Review) and were meta-analyses, systematic narrative reviews of evidence-based studies, integrative reviews of theories on SMs, and classical reviews on CB. The information was coded in Excel for subsequent analysis and discussion. The chosen criteria were: (a) type of document, (b) authorship, (c) year of publication, (d) title, (e) journal or book in which the results were published, (f) country in which the research was conducted, (g) objectives of the study, (h) study design, (i) sample size and composition (N and/or K), (j) data collection instruments, (k) language of publication, and (l) main results. Table 1 lists the most relevant characteristics of the included studies.

**Table 1:** Studies and characteristics of the records included in the systematic review.

**Type of document**	**Years**	**References**	**Explanatory factor**	***N***	***K***	**Dependent variable**	**Main results**
Meta-analysis	2007–2019	Agostini and van Zomeren (2021)	Relative deprivation Identity Effectiveness Moral conviction System justification Beliefs	123,707	403	Attitudes, behavioral intentions and collective informed behavior	Confirms the strong effects of the 4 motivational factors of participation in collective action and SM
	2011–2020	Akfirat et al. (2021)	Identity movements network	18,242	46	Attitudes, behavioral intentions and collective informed behavior	Confirms that social identity is associated with a medium effect size on participation in network-based CB and SM
	Up to 2020	Jahnke et al. (2021)	Identity Relative deprivation Threat Symbolic or moral		Betwee*n* 10 and 30	Attitudes, behavioral intentions and informed violent political behavior	Concludes that RD, identity, symbolic or moral threat are associated with intentionally violent political behavior.
	1961–2010	Smith et al. (2012)	Relative deprivation	49,242	99	Intention, attitude and informed extra-institutional collective behavior	Confirms that RD is associated with a medium effect to CB—among other variables.
	1961–2016	Smith et al. (2018)	Relative deprivation	200578		It includes 303 effect sizes from 231 different nations and measures of individualism and distance to power.	Concluding that the association between RD and CB is greater in countries with an individualistic culture.
	Up to 2008	van Zomeren et al. (2008)	Relative deprivation Identity Efficiency	Between 10,051 and 15,805	Between 64 and 65		Confirms the strong effects of the 3 motivational factors of participation in collective action and SM
Classic review	Up to 1969	Milgram and Toch (1969)	SM and CB				Reviews CB patterns questioning unanimity beliefs and behavior, and describes SM.
	1983	McPhail and Wohlstein (1983)	CB				Reviews CB patterns questioning unanimity of beliefs, behavior and defines collective meetings, CB, riots, rallies, etc.
	Up to 1994	Snow and Oliver (1995)	SM and CB				It critically reviews theories of CB based on masses and people without identity and describes developments in the theory of resource mobilization and its emphasis on organizations, rational action, the importance of social networks, and the narrative frameworks approach to collective action. It discusses the evidence that supports and limits these explanations of CB and SM.
Recent review	Up to 2014	Reicher and Drury (2015)	CB in disasters, manifestations and collective rituals from the point of view of social identity theory and self-categorization (SCT).				Questions the predominance of panic and selfish behaviors and postulates SCT-based explanation of CB
	Up to 2019	Drury and Reicher (2020)	CB similar to previous review				Reviews Anglo-Saxon studies CB questions irrationality and purposeless violence
	Up to 2019	Drury (2020)	CB similar to previous review				Idem—Review on CB in disasters, demonstrations and collective rituals from the point of view of social identity and SCT theory
Evidence-based systematic review	Up to 2012	van Stekelenburg and Klandermans (2013)	Explanatory factors for the emergence of SM				Reviews SM explanatory factors such as resource theory for mobilization, RD, collective identity, emotions and social networks.
Evidence-based systematic review and referral to meta-analysis	Up to 2012	van Stekelenburg et al. (2013)	Explanatory factors of SM, based on meta-analysis (van Zomeren et al., 2008)				Systematic review and synthesis of the above-mentioned explanatory factors and the development of SM, based on the meta-analysis of van Zomeren et al. (2008)
Narrative review of long-term effects	1967–2015	Vestergren et al. (2017)	Participation in SM	57Artics 39 in the U.S. and only two from non-Western nations			Narrative systematic review of the effects of SM participation based on dozens of articles. Shows positive effects on emotionality, identity, social integration, ideological anchoring, and empowerment, but also negative effects such as burnout and negative effects of SM participation that fail.
Review of epidemiological studies impact on mental health	Up to 2018	Ni et al. (2020)	Effect of CB on public and demonstrators' mental health	57,487	52		Studies found a 7% risk increase in depression after CB 6 longitudinal studies Not related to direct participation in CB

RD, Relative deprivation; SCT, self-categorization theory.

##### 2.1.1.5. Data synthesis

The years of review and number of studies or *K*, samples or *N*, and effect sizes are described above.

Conceptualization and measurement of variables and effect sizes are discussed when examining the different psychosocial explanatory factors for SMs participation (see next Tables 1–7 and Figures 1–9). All meta-analyses used correlation as the effect size and did not overlap in years or types of studies.

## 3. Results

Synthesizing the studies reviewed, we first present how participation in SC and SMs is measured, the patterns found in CBs, psychosocial factors for participation in CB and SMs, and the long-term effects of participation in SMs. See the glossary defining motivational constructs in the Appendix 1.

### 3.1. How participation in CB and SMs is measured

The review of systematic studies and meta-analysis found that CB studies use as indicators the following questions: (a) Intentional sabotage of work performance; (b) willingness to block a road, willingness to block bulldozers, or set up barricades; (c) willingness to approve civil disobedience; (d) willingness to sign petitions and join strikes; and (e) participation in self-reported riots (Smith et al., 2012) or attitude in favor of violent political action (Jahnke et al., 2021). Observational CB data were used in the past and are now rarely used (Drury, 2020).

More general instruments, not limited to protest CB or demonstrations related to socio-political demands, have also been developed to measure the frequency of collective encounters. Participants are asked to express the frequency of their participation in nine major types of collective gatherings in which people usually participate (e.g., “How often do you attend ceremonies or social gatherings?”), among which were included (1) family gatherings, (2) lunches or dinners, (3) concerts or musicals, (4) movies, (5) union meetings, (6) neighborhood meetings, (7) community meetings, (8) party meetings, (9) association meetings, and (10) attendance at public religious rituals. For frequency of attendance, the responses ranged from 0 (*never*) to 4 (*more than once a week*) (Cusi et al., 2022) (see Appendix 2).

Social psychology SM studies operationalize these as (a) attitudes toward collective action (e.g., “being a supporter of collective action”), (b) intentions or action tendencies to participate in collective action (e.g., “willingness to participate in collective action”), (c) self-reports on past behavior (e.g., “the number of petitions signed last year”) or actual behavior (e.g., “sign a petition to improve the current situation of XXX in Y”, “participate in a demonstration”) (van Zomeren et al., 2008; Agostini and van Zomeren, 2021) (see Appendix 2).

Population survey studies, such as the World Values Survey (WVS) or the European Social Survey (ESS) collect data on the differences between SMs and CB participants and non-participants. For example, the World Values Survey asks about participation in “legal' or institutional and “illegal” or extra-institutional social mobilizations. Its questions are “Indicate for each of the following actions, whether you have done them, would be willing to do them, or would do them under no circumstances”. “Have you participated in the past year (or are you willing)” to (a) sign a petition; (b) participate in a boycott; (c) participate in a legal demonstration; (d) participate in a legal strike; (e) participate in an illegal strike; (f) occupy buildings or factories, via three response alternatives “Has done,” “Could do it,” and “Would never do it”. These studies provide little information on the dynamics of demonstrations and their effects. The protest survey method advocated by Klandermans and colleagues consists of surveying a large number of demonstrators during a protest and, at the same time, recording the characteristics of the context, the police, and the mobilizing actor (van Stekelenburg et al., 2018).

### 3.2. Patterns of CB

A cross-cultural study surveyed student samples from 40 countries on participation in seven specific types of group gatherings, finding that the vast majority (87%) attended three or more types of events per year. The mode was four types of events. “Concerts, dance performances or musicals” were attended by 92% once a year; “festivals” (music, art, etc.) by 81%; “local folk/folklore events” by 73%; “religious events” (i.e., regular religious services, except baptism, marriage and funeral services) by 59%; “sporting events” (soccer and football) by 43%; “street demonstrations” by 31%; and “large religious ceremonies”, such as Fatima, Lourdes, Czestochowa, and Mecca, by 24%. The results show that most people never go alone to any type of event, except for normal religious events. People are more likely to always participate in religious events with family members than in any of the other types of crowded events. However, at all types of events, the majority of respondents always or sometimes attend with family members. The importance of friendship emerges for one-third of the respondents at the other types of events, who say that they always attend events with friends. Attending with acquaintances is less frequent, even though the majority of the sample claims to do so always, often, or rarely. For leisure, recreational, and less ideologically charged collective gatherings, such as concerts, festivals, and folkloric and sporting events, the reasons for participating were because they liked the activity and to share and experience happy moments. For religious collective gatherings and demonstrations, “to have a better life” is more often invoked as a reason to participate (Cintra Torres et al., 2018). Another study inquired about the frequency of participation in collective gatherings. It found two dimensions, one of the large gatherings similar to the previous ones (political, union, neighborhood, and community) and another of gatherings with friends and family. The mean attendance at large meetings was lower (*M* = 0.91) than that of informal meetings (*M* = 2.28, range 0 never to 4 very frequent). Both types of encounters were associated with collective effervescence (Cusi et al., 2022).

Next, we will review the behavioral, cognitive, and emotional patterns or regularities of CB. These studies, although observational and descriptive in nature, are important because, first, they provide information that questions and clarifies psychosocial explanations of SMs. Second, the review of the outcomes of CB or positive effects of participation in demonstrations and collective gatherings on identity, emotions, and social integration serves as support, and as a microsocial explanation, of the medium- and long-term psychological effects of participation in SMs.

### 3.3. CB and the myth of excessive emotionality and the illusion of unanimity of beliefs and behaviors

Earlier systematic observation studies (McPhail and Wohlstein, 1983) and older and recent studies using *in vivo* interviewing of demonstration participants (Milgram and Toch, 1969; van Stekelenburg et al., 2018) challenged that CBs were characterized by unanimity of beliefs, identification, and behavior, as well as pervasive emotionality.

Four studies, which sought to contrast the hypothesis that sharing a common set of beliefs was a necessary condition and was associated with participation in mass demonstrations, found that a dispersion of beliefs was prevalent (Milgram and Toch, 1969; Quarantelli and Hundley, 1969; Marx, 1970; Stallings, 1973; Ladd et al., 1983). These studies concluded that an ideological consensus is not a necessary precondition for participation in collective action. In doing so, they criticized Smelser's “generalized beliefs” and, in a modernized but still similar version, the dominant collective action frames approach to SMs (see below).

A study of 81 demonstrations, including collective rituals such as gay pride on May Day, questioned whether there was homogeneous collective identification: two-thirds of the demonstrators were found to identify with the organizers or the SMs and three-fourths with the other demonstrators or participants in that collective behavior. Three-fifths were identified with the organizers and the participants, one-fifth only with the other protesters, and less than one out of 10 only with the organizers. In addition, one person out of six did not identify with either the organizers or the protesters (Klandermans and van Stekelenburg, 2019). Systematic observations of spontaneous collective behavior have shown that only in a proportion of <1%, all participants acted in the same way (Milgram and Toch, 1969). Sequences of action indicating the existence of unanimity in crowds are never observed since participation in activities is sequential rather than simultaneous. More generally, three patterns of action can be identified: certain actions (singing and making certain gestures) are preceded by a suggestion by an organizer; others are generated interdependently through consultation or interaction between close individuals (conversations, formation of small groups, which are particularly visible in the phases before and after the procession); finally, certain actions are initiated independently by individuals at more or less the same time [e.g., cheers and applause (Filleule and Tartakowski, 2013)]. Concerning the perception of expressive and emotional behavior, cheering and excitement characterize a limited time of a typical manifestation (McPhail and Wohlstein, 1983). However, studies found that collective effervescence or intense emotional shared activation is common and related to positive outcomes, including increased creativity (Castro-Abril et al., 2021) (see Appendix 2).

### 3.4. CB and disasters: the myth of panic and collective resilience

Studies show that looting in disasters is rare (see Table 2). When they do occur, it is most often a reflection of existing social forces, not a collapse. Appropriate or “pro-social” behavior is more likely to emerge—people take food or fuel as a way of helping themselves. A collective resilience or predominance of rational, altruistic, and solidarity actions is generally manifested after catastrophes, in addition to the absence of mass panic and riots (Quarantelli and Dynes, 1977; Drury et al., 2019). This euphoric, altruistic community, heroic phase and “honeymoon” stage, as it has been called, in response to catastrophes, is characterized by: (i) increased internal solidarity, (ii) a sense of unity, (iii) the disappearance of community conflicts, (iv) a mood of democratic utopia, and (v) a general sense of altruism and heroic action (Matthewman and Uekusa, 2021). This altruistic “compassionate” stage usually ceases after a few weeks. Moreover, this internal solidarity response does not occur in all situations, nor does it eliminate social differences and conflicts (Páez and da Costa, 2022). Immediately after the impact of a catastrophe or traumatic event, there is an emergency or heroic phase that lasts 2–3 weeks. This phase is characterized by high anxiety, intense social contact, and repetitive thoughts about what has happened, followed by the second phase of inhibition, which lasts between 3 and 8 weeks. This phase is characterized by a significant decrease in the frequency of expressing or sharing socially about what happened. People seek to talk about their difficulties but are “burned out” from listening to others talk. In this phase, anxiety and arguments increase, and people choose to avoid talking, followed by silence and a “burnout” from talking about the issue. Finally, 2–4 months after the catastrophe, the level of talking and thinking converges and decreases, producing an assimilation of the event in the general collective and a return to routine functioning—this refers to people not directly affected (Pennebaker and Harber, 1993; Rimé, 2020).

**Table 2:** Explanatory factors of CB and SM.

	**Relative deprivation**	**Collective efficacy or efficiency**	**Collective identity**	**Emotions**	**Convictions and moral beliefs**	**Ideology and system justification beliefs**
Why people take part in SM?	Because they are aggrieved as a group	Because they perceive their group can change the environment	Because they identify with the group	Anger, affective RD Hope and positive emotions Perceive Emotional climate favorable to SM	Their moral beliefs have been challenged and feel morally obligated	They do not believe that the social system is fair and people do not get what they deserve
Who are the participants?	People altered by unfair ingroup treatment	People with High political Efficiency High collective efficacy Distrust institutions	Identified with group, class In particular with emergent politized identity	People feeling anger but also pride and hope	Even if they are not part of the group aggrieved, they feel a moral obligation to mobilize	People disagree with system justifications beliefs In conservative SM people share beliefs that social system is fair

### 3.5. CB in riots: the myth of blind violence discharge

The relationship between collective encounters and violence has also been examined. Observational studies found that most CB was non-violent—in US samples in general (McPhail and Wohlstein, 1983). A study of major protests in the 21st century, which analyzed in-depth 2,809 protests occurring between 2006 and 2020 in 101 countries covering more than 93% of the world's population, confirmed that the dominant forms of protest were nonviolent: they were demonstrations or marches in 61% of the cases and assemblies in 59%. In a significant minority, there were more violent forms of protest: barricades in 22%, occupations in 21%, looting/vandalism in 19%, and violence in 15% (Ortiz et al., 2022). Postmes and Spears (1998) conducted a meta-analysis of 60 studies of deindividuation, a state supposedly induced by being in a collective gathering or crowd, confirming that it provokes anti-normative or violent behavior although not always—and the association was shown to be weak (*r* = 0.09). These experiments confirmed that larger group size and anonymity to the out-group, i.e., responses being anonymous to the experimenter or to the people who are the victims of the anti-normative behavior, are associated with greater aggression or deviant behavior. Experimental studies also found that reduced public self-awareness (i.e., being aware that one is present or in front of others) is also associated with more aggression or deviant behavior. Field studies confirmed the effect of group size on lynchings in the US, with the larger the crowd size, the more heinous and brutal the aggression (Leader et al., 2007). The results reviewed by Postmes and Spears (1998) suggest that aggression and antisocial behavior are not inevitable by-products of situations analogous to being in a crowd, of anonymity, and the presence of many people. When norms and standards promote aggressive behavior (e.g., being dressed in uniform or Ku Klux Klan-style clothing that may trigger norms associated with fighting and aggression), antisocial behavior is facilitated. However, when norms and standards promote positive behavior (e.g., nursing uniforms that are associated with norms associated with caring and helping), pro-social behavior was facilitated. In other words, deindividualized behavior increases adherence to situationally salient norms. Overall, it has been concluded that individuals in a “crowd situation” act as a function of their salient collective identity (e.g., “nurses”) and social norms (e.g., “one must help”) (Reicher et al., 1995).

Studies on crowds and collective violence draw three major conclusions (Allen, 1970; Drury, 2020). First, people are predisposed to riot when they have a sense of being treated illegitimately and of the futility of making polite complaints or conventional protests. Second, the events that initiate riots embody these beliefs, but they also unite people, give them a sense of shared outrage, and empower them to fight back. Third, riots themselves are not mindless explosions in which anything is possible. Rather, the behavioral patterns of the crowds reflect the protesters' worldview: their sense of who is a friend and who is a foe. For example, in the St. Paul's riots in Bristol, England, which occurred on 2 April 1980, only those perceived to be inimical to St. Paul's identity were attacked, primarily by the police. Second, there were defined geographic boundaries: police were only attacked while they were within St. Paul's boundaries and were left alone once they left (Drury and Reicher, 2005).

Studies also suggest that collective violence follows a logic of moral legitimacy. Analyses of ethno-racial collective violence (such as lynchings) showed that participants in violence against people of different ethnicity or race and “inferior” status, including brutal crimes, such as the lynching of alleged rapists or snitches or simple scapegoats, or the killing of children and the elderly in ethnic riots, were carried out by members of semi-organized groups and not mobs, who believed in the morality and justice of their action. These actions of collective violence functioned as a means of social control by indicating to the members of the attacked groups that their marginal status and position were “real”—regardless of official legal changes. The torture, murder, and public mutilation of lower-class black males accused of sexually assaulting white women were not only aimed at restoring the purity of white women and the “moral integrity” of that race but also served to maintain the hierarchy between whites and blacks (Javaloy et al., 2001, 2007; Leader et al., 2007; Páez and da Costa, 2022). Indeed, it has been posited that collective violence against people labeled as deviants (e.g., people who violate religious rules) originates from fear, justified anger, and punitive desire for retaliation stemming from violations of moral imperatives (Asif and Weenink, 2019).

Recent CB reviews (Reicher et al., 1995; Drury et al., 2012; Drury and Reicher, 2020), developed by social psychologists framed in the SCT tradition, have emphasized that crowd formation and collective behavior are underpinned by the development of a shared social identity whereby people see themselves and others in terms of belonging to a common category. This results in three psychological transformations: members perceive the world in terms of collective values and belief systems; they coordinate effectively; and, therefore, they are empowered to realize their collective goals. These transformations explain the social form of crowd action. At the same time, the acts of crowds are intergroup phenomena. It is through the intergroup dynamics between the crowd and an external group (usually the police or other opposing groups of protesters) that the social identity of crowd members can change through the way outgroups understand and respond to their actions, reinforcing or resulting in the creation of a collective identity (Drury and Reicher, 2020).

### 3.6. CB and wellbeing

Reviews of studies show that public and multitudinous religious or secular rituals (in India, in Mecca, etc.), including collective encounters of positive valence and without ideological or vindictive charge, such as folkloric parades, can reinforce wellbeing, empowering people, increasing collective identity and self-esteem (Drury, 2020), fusion with the group (Henríquez et al., 2020), increasing social cohesion (Hawdon and Ryan, 2011), and reinforcing agreement with values and beliefs, with effect sizes between *r* = 0.20 and 0.30 at least in the short-medium term (Páez et al., 2015; see Pizarro et al., 2022 in this issue). A systematic review states: “Festivals provide economic, social, and cultural benefits to the communities in which they occur” (Tanford and Jung, 2017, p. 209). Festival observance was associated with low psychological distress in a study in Japan. Festivals provide entertainment and cultural interaction in the community and strengthen social integration or social capital, which reinforces wellbeing (Minamizono et al., 2013). Partially confirming the positive effects of participation in collective gatherings, a review of 49 longitudinal studies confirmed that participation in public religious activities (going to mass) weakly predicted good mental health, *r* = 0.08 (Garssen et al., 2020).

### 3.7. Conclusion: CB studies and psychosocial theories of SMs

CB studies have implications for psychosocial theories or explanatory factors of SMs (see below), like collective action frames, rational logic of collective action, collective identity, moral convictions, and emotions. Studies on patterns of CB questions, partially, collectively frame explanations of SMs, because of a lack of evidence of generalized beliefs. Second, CB in catastrophes and riots shows that people and rioters act in an adaptive or functional goal-oriented way. Evidence on CB during riots and catastrophes supports explanations of SMs by the rationality logic of collective action (Klandermans, 1984). Collective identity played a prominent role in CB patterns during the riots. Moreover, the approach based on Tajfel and Turner's theory of social identity was applied to catastrophes and riots, and a Social Identity Model of Crowd Behavior was developed (Drury et al., 2019). The review of violent collective behaviors, even the most despicable ones, such as lynchings, suggests that they follow a moral logic—supporting the importance of moral convictions as an explanatory factor of SMs. Finally, although moments of intense emotionality are limited, collective effervescence or shared emotional activation is associated with greater creativity and empowerment. These results of the CB studies support the theories that emphasize the role of emotions in SMs.

### 3.8. Factors of participation in SMs and CB

The explanations that have been given in temporal order about the motives that lead to participation in SMs and CB are presented. The following table, inspired by van Stekelenburg and Klandermans (2013) and Páez and da Costa (2022), synthesizes the explanations reviewed by meta-analyses of SMs. For the approaches of opportunities and resources mobilization, rational action, expectations, and motives for action, as well as collective action frameworks, no meta-analyses were found and are examined below (see Table 2).

### 3.9. Relative deprivation, grievances, and injustice

**Figure 2:**
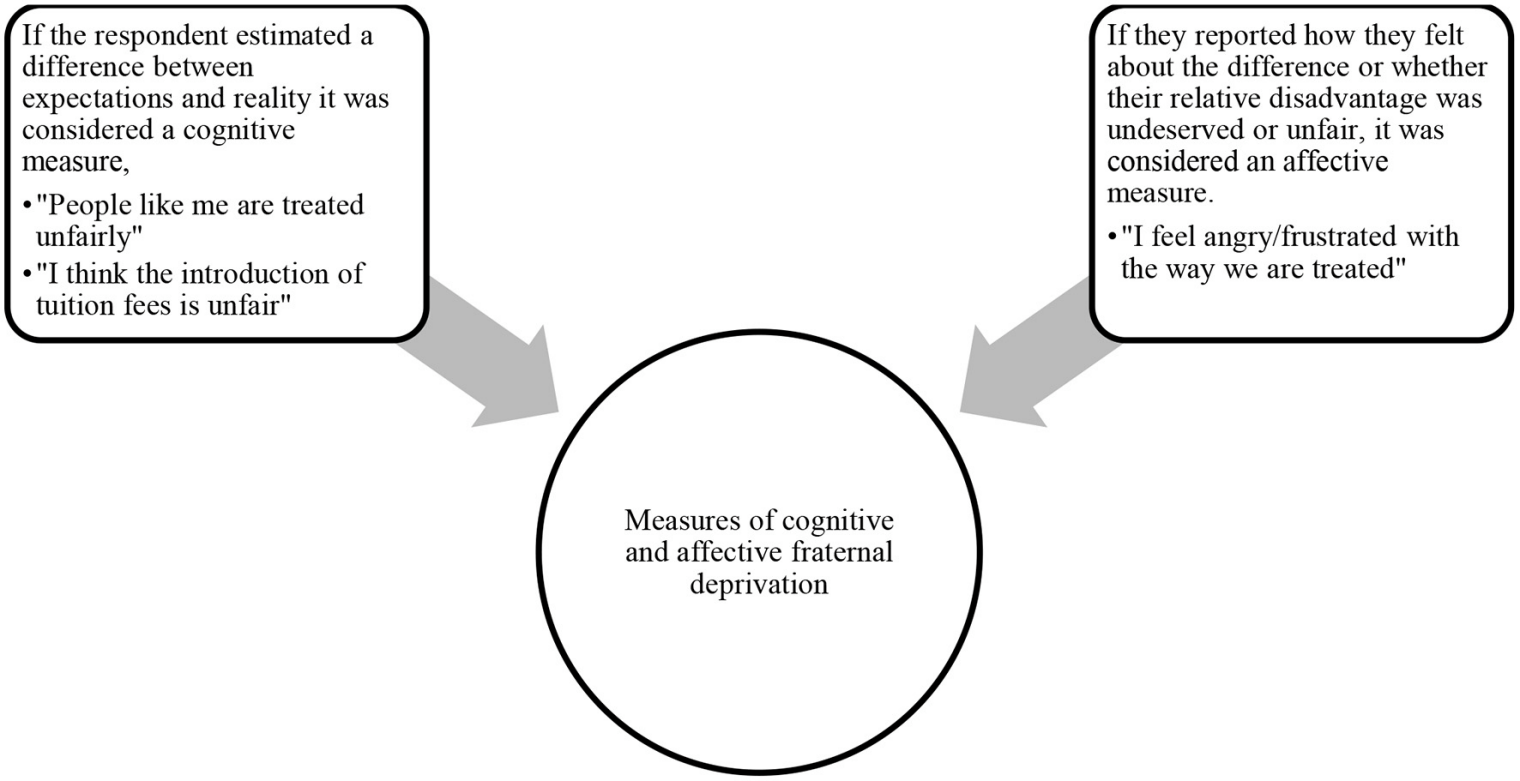
Measures of relative deprivation (Agostini and van Zomeren, 2021).

Relative deprivation (RD) theories attempted to explain the causes of the feeling of discontent or dissatisfaction, which can eventually lead to collective action and the development of SMs, and were developed in the 1960s. This explanation holds that people evaluate what they have in comparison with what they believe, in fairness, they should have. If they get less than they expect, they consider it unfair and discontent spreads among them. RD is linked to Tocqueville's idea of the circumstances in which revolutions arise. According to this classic 19th-century French essayist, revolutions do not occur in periods of decline or stagnation—stable and permanent misery produces despair rather than rebellion. Collective protest behaviors occur when, after a period of improvement, the situation worsens. Four key features of the experience of RD are as follows: first, people must care about what they lack. RD involves both wanting and deserving. Second, people must believe that the current situation is unlikely to change without intervention. Otherwise, the possibility of improvement may temper anger and increase hope for the future. Third, people should not see themselves as responsible or to blame for the deprivation. Fourth, people should see the process that produced the deprivation as illegitimate. However, these appraisals have rarely been measured independently in research (Agostini and van Zomeren, 2021). A distinction is made between personal RD (i.e., the perceived discrepancy between personal expectations and achievements) and fraternal or collective RD (i.e., the perceived discrepancy between what our group has compared to others). Studies show that personal frustration or deprivation leads to apathy rather than mobilization. What motivates participation in collective behavior is fraternal or group-focused deprivation. People who perceived that their social group (not themselves individually) received less than they expected or thought they deserved, relative to other groups, were those who showed the greatest tendency to social mobilization—for example, French-speaking citizens of Quebec relative to English speakers (Smith et al., 2012). See Figure 2 for operational measurements of RD.

Affective RD measures address judgments related to the person's situation including measures of unfairness, negative mood, frustration, discontent, dissatisfaction, anger, and resentment. The purely cognitive measures asked respondents to estimate differences between their current situation and a comparison with a referent—such as another group (Smith et al., 2012). The relationship between fraternal RD and participation in mobilizations or CB has been corroborated by van Zomeren et al. (2008) and Agostini and van Zomeren (2021), and by another synthesis of studies, although in this meta-analysis, the relationship is weak to medium, at *r* = 0.15 (Smith et al., 2012). Another meta-analysis (Jahnke et al., 2021) examined the relationship between relative group deprivation and participation in violent political action in youth—which in part is shaped by violent CB. In this meta-analysis, the favorable attitude or willingness to engage in violent political action was the dependent variable rather than actual violent behavior. Based on 11 studies, it found a correlation of *r* = 0.19 between relative deprivation and participation in violent political action—similar to the result of Smith et al. (2012). The smaller effect size of Smith et al. (2012) and Jahnke et al. (2021) is probably explained by the fact that the criterion variable was extra-institutional and more violent CB.

According to two reviews (van Zomeren et al., 2008; Agostini and van Zomeren, 2021), the association of RD with SMs or collective actions is stronger with affective deprivation (*r* = 0.49 and *r* = 0.39) than with cognitive deprivation (*r* = 0.33 and *r* = 0.34), a finding corroborated by Smith et al. (2012) meta-analysis (*r* = 0.17 vs. = 0.077, Smith et al., 2012).

Meta-analytic integration of meta-analysis was performed using CMA 3.0 and used *N* to weigh each study. This analysis is helpful to have a global effect size estimation—even if we are not working with individual studies and effect sizes—and the procedure has limitations. The following dashboard describes results based on *N* = 284,254 and *k* = 687 studies. An effect of deprivation/injustice on SM participation is found—random model *r* = 0.296 CI 0.13–0.45. Based on *k* = 216 studies, an effect of cognitive deprivation/injustice on SM participation is found—random model *r* = 0.25 CI 0.055–0.43 on *r* in bold is the weighted estimate. Jankhe's meta-analysis was excluded because some criterion variables were not CB (see Table 3).

**Table 3:** Relationship of relative deprivation and injustice with SM and CB.

**Meta-analysis**	**Factor**	**Years**	***N***	***K***	** $\bar{r}$ **
van Zomeren et al. (2008)	Injustice deprivation	Until 2007	15,855	65	0.35
	RD cognitive			38	0.34
Smith et al. (2012)	Deprivation with collective behavior	From 1961 to 2010	49,242	99	0.148
	RD cognitive			60	0.077
Agostini and van Zomeren (2021)	Injustice deprivation	2008 up to the present	82,326	329	0.38
	RD cognitive			108	0.33
					$\bar{\text{r}}$ **=** **0.296**

RD, relative deprivation.

Values in bold indicates weighted mean effect size.

Explanations of participation in SMs due to relative deprivation and comparative grievances were questioned since grievances are not a sufficient reason to participate in SMs. Indeed, grievances abound while protest does not. Therefore, why do some aggrieved people mobilize and others do not?

### 3.10. Collective and political effectiveness

**Figure 3:**
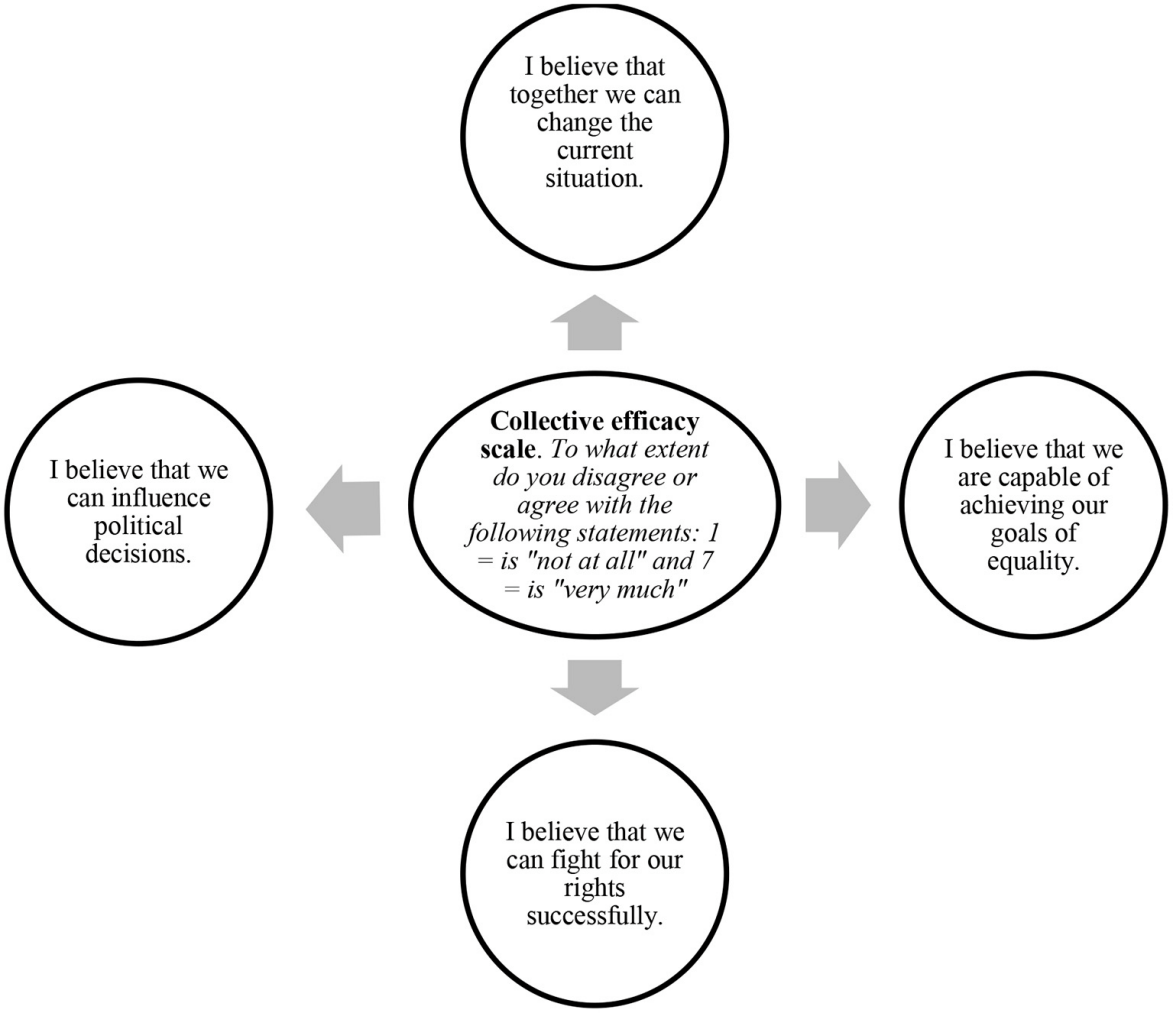
Measures of collective efficacy. Source: van Zomeren et al. (2010) and Zumeta et al. (2020).

Another social-psychological answer to the question of why some people mobilize and others do not is efficacy. To what extent do people expect group-related problems to be solved through joint efforts? For the perception of the possibility of change to take hold, people must perceive that the group is capable of uniting and that the political context will be receptive to their group's demands. The first refers to group efficacy: the belief that group-related problems can be solved through collective efforts; and the second refers to political efficacy: the sense that political actions can have an impact on the political process. Political efficacy is conceptualized with two dimensions: internal efficacy, or the extent to which someone believes they understand politics and therefore participates in it; and external efficacy: citizens' faith and trust in government. Negatively related to political efficacy is political cynicism—defined as the opposite of political efficacy and inversely related to trust in government. van Zomeren et al. (2008) identified perceived efficacy as a main predictor of collective action, that correlates strongly with collective behavior (“people participate in collective action if they believe that this will make it more likely that the relevant goals will be achieved”, p. 506). See Figure 3 for operational measurements of this conceptual variable.

Based on *N* = 69,542 and *k* = 154 studies, we find an effect of efficacy on participation in SMs—CMA 3.0 randomized model *r* = 0.356, CI 0.33–0.34 (see Table 4). This process can be illustrated by the studies on Basque radical nationalists in the 80s and 90s of the last century who supported illegal mobilizations, compared with people living in the Basque Autonomous Community who were not in favor of them. The former was characterized by (a) a negative perception of institutional political channels; (b) perception of high political efficacy or internal control of the environment; that is, they perceived themselves as having socio-political resources but institutionally blocked, which legitimized extra-institutional action. They valued the efficacy of their participation more highly, had higher expectations of the participation of others in mobilizations, and believed more in the probability of their success than people who did not participate in mobilizations (Valencia, 1990).

**Table 4:** Efficiency relationship with SM and CB.

**Meta-análisis**	**Factor**	**Years**	***N***	***K***	** $\bar{r}$ **
van Zomeren et al. (2008)	Efficacy	Until 2007	12,758	53	0.34
Agostini and van Zomeren (2021)	Efficacy	2008 up to the present	56,784	101	0.37
				$\bar{\text{r}}$ **=** **0.365**

Values in bold indicates weighted mean effect size.

### 3.11. Collective identity

**Figure 4:**
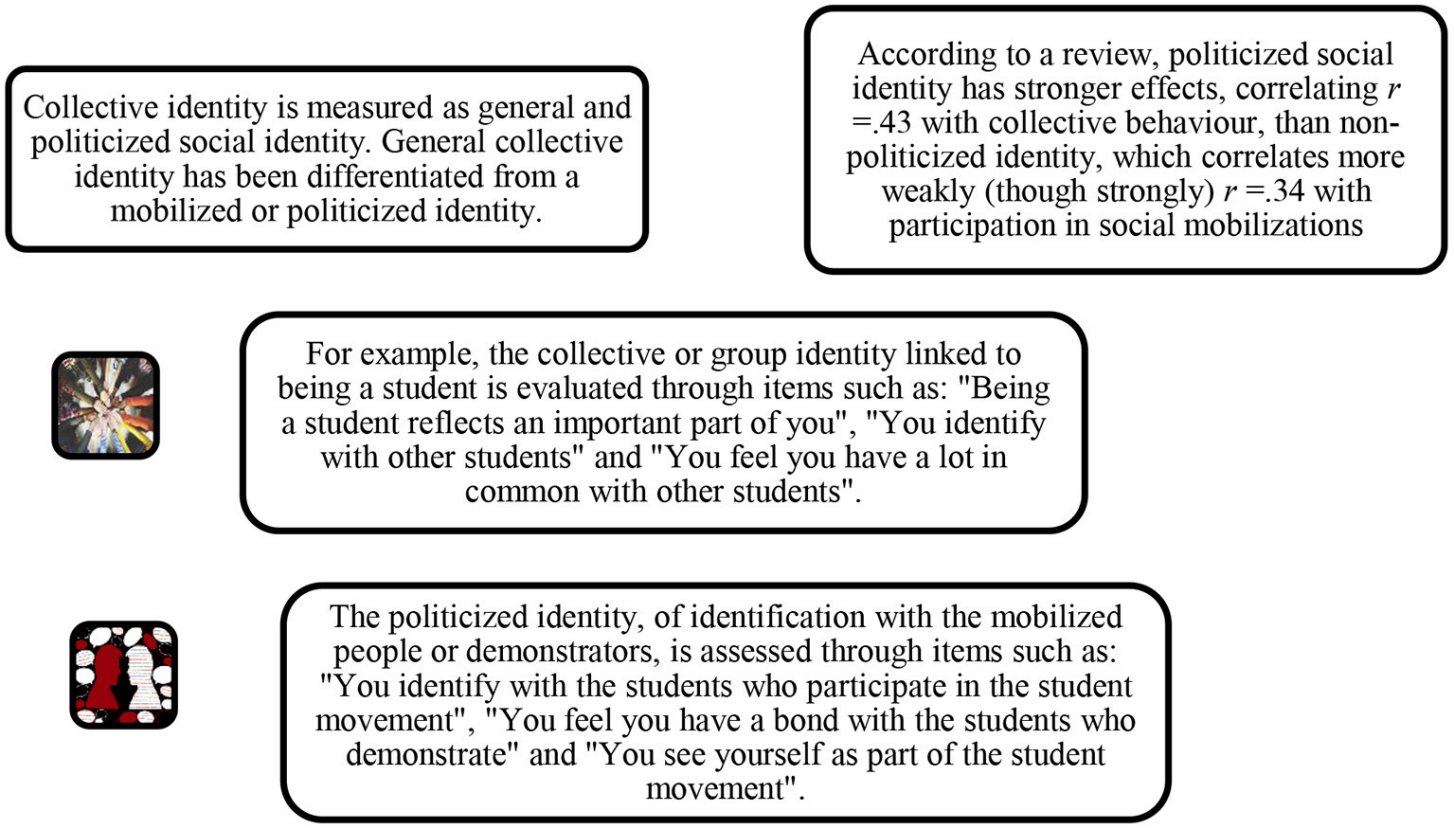
Measures of collective identity. Source: van Zomeren et al. (2008).

In the 1980s, it became clear in SMs research that the instrumental logics of grievances and efficiency are not sufficient reasons to participate in a protest. Increasingly, the importance of collective identity as a factor stimulating participation in a protest was highlighted. Sociologists argue that the generation of a collective identity is crucial for a movement to emerge (Touraine, 1978). Similarly, social psychology studies concluded that the more people identify with a group, the more inclined they are to protest on behalf of that group. This relationship has also been confirmed by three meta-analyses (van Zomeren et al., 2008; Agostini and van Zomeren, 2021; Akfirat et al., 2021). van Zomeren et al.'s (2008) and Agostini and van Zomeren (2021) review found that social identity predicted participation in collective action, correlating with collective behavior (“if group members perceive the intergroup status difference to be illegitimate and unstable, they are more likely to identify with their group and participate in collective action to change the intergroup status difference”, p. 507). See Figure 4 for operational measurements of this conceptual variable.

A meta-analysis examined the role of identity in social network-based mobilizations or large-scale collective actions that are known to mobilize overwhelmingly through digital platforms (e.g., the Occupy Wall Street movements, the Arab Spring, the Yellow Vests in France, and the social outburst in Chile). It has been argued that, compared to conventional SMs, network-based collective action is much more personalized, and its underlying psychological mechanism does not require the symbolic construction of a united we. These digital connections are made based on interpersonal relationships such as friendship or family. Participants in an action coordinated by networks would not need to develop a shared ideological framework to establish connections (Akfirat et al., 2021). Therefore, these digital movements differed significantly from traditional collective actions in terms of the characteristics of the organizations that developed (newly created, without formal membership, and with scarce resources). However, contrary to the idea that collective identity is less relevant in mass movements in the digital era, the results of the meta-analysis by Akfirat et al. (2021) revealed that there is a strong relationship between social identification and participation in collective action, *r* = 0.45. The relationship between collective action participation and identification with emergent groups was also found to be stronger than identification with pre-existing groups, of *r* = 0.52 and *r* = 0.34, respectively. Identification with an emerging group (e.g., protest groups and opinion groups) better predicts participation in collective action (Agostini and van Zomeren, 2021; Akfirat et al., 2021; pooled *r* = 0.48) than identification with pre-existing social groups (e.g., nations, religious groups, and ideological groups; pooled *r* = 0.34). The meta-analysis by Jahnke et al. (2021) that examined the relationship between identity and participation in violent political action, based on 11 studies, found a correlation of *r* = 0.21. This smaller effect size reaffirms the idea that motivational factors are more associated with institutional SMs and CB than with riot or violent behavior.

Based on *N* = 105,608 and *k* = 439 studies, a strong effect of collective identity on participation in SMs was found—random model *r* = 0.41, CI 0.37–0.45 (see Table 5). The meta-analysis by Jahnke et al. (2021) was excluded from the final calculation for the reasons described above.

**Table 5:** Relationship of identity with SM and CB.

**Meta-análisis**	**Factor**	**Years**	***N***	***K***	** $\bar{r}$ **
van Zomeren et al. (2008)	Identity	Until 2007	10,051	64	0.38
Akfirat et al. (2021)	Identity movements network	2011 until 2020	18,242	46	0.45
Agostini and van Zomeren (2021)	Identity	2008 up to the present	77,315	329	0.40
				$\bar{\text{r}}$ **=** **0.41**

Values in bold indicates weighted mean effect size.

**Figure 5:**
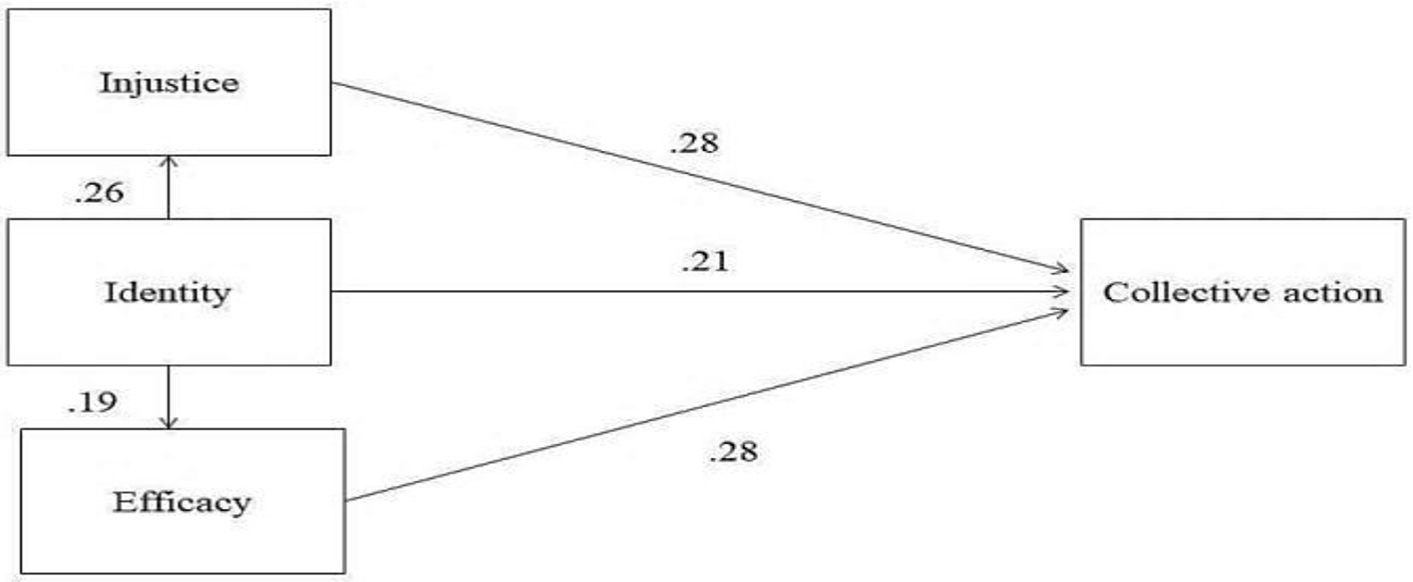
SIMCA model. Source: van Zomeren et al. (2008).

The Social Identity Model of Collective Action (SIMCA) that was developed from the meta-analysis of van Zomeren et al. (2008) postulated that each of the motivations (RD or injustice, identity, and efficacy) has a unique effect on collective action and that social identity plays a central role in mediating between efficacy and injustice (see Figure 5).

### 3.12. Emotions and SMs

Factors that are embedded in frames of reference of identity, injustice, and efficacy (see discussion of collective action frames below) will activate and motivate people if they have an emotional impact (as already suggested by the fact that relative affective deprivation is more important than cognitive deprivation). Recently, since the end of the 20th century, personal and collective emotions have been included as factors explaining social mobilization (Bou Zeineddine and Leach, 2021).

Injustices exist permanently in society and people, even though they are aware of them, do not mobilize. The emotions of indignation and anger will fill the framework of injustice with motivational content. Anger is considered the prototypical protest emotion (van Stekelenburg and Klandermans, 2007). Group anger can be conceived as an emotion that responds to the perception that there is an illegitimate negative event or a breach of norms, made intentionally by another group, a positive evaluation of one's resources and perception of control, and is associated with the tendency to attack. Anger reinforced the mobilization of peasants for their situation in Holland and Spain (Sabucedo et al., 2017). Indignation is righteous anger at something wrong, bad, and immoral and moral outrage is anger at the violation of a moral norm. Moral outrage shares with anger the perception of injustice or oppression that is a moral transgression, although it differs from anger because the injustice or wrong is not necessarily experienced personally. Some studies suggest that being impacted by immoral events (such as corruption of power, in the case of Spain 15-M) activates moral anger, which reinforces participation in SMs (Sabucedo et al., 2018). There is also a relationship between anger and efficacy: people who perceive the ingroup as strong are more likely to experience anger and desire to act out; people who perceive the ingroup as weak are more likely to feel fear and withdraw from the outgroup. Using relative deprivation or affective injustice as a proxy for anger and as an indicator of the influence of negative valence emotions on participation in social protest, a very strong estimate of its importance is obtained (see Figure 2 for affective RD measures and Table 6). The emotional experience of group-based relative deprivation is more strongly related to collective action than its perception because the emotional experience of injustice (e.g., anger) reinforces the motivation to act, and the perception of injustice is central to emotions such as anger. In line with this, van Zomeren et al. (2008), Smith et al. (2012), and the meta-analysis of Agostini and van Zomeren (2021) found that felt injustice produces a larger effect than perceived or cognitive injustice. It should be mentioned that the effect size of affective RD on collective behavior was smaller in Smith et al. (2012) than in the two meta-analyses on SMs. In the same vein, the meta-analysis by Jahnke et al. (2021), which examined the relationship between negative intergroup emotions and participation in violent political action, based on nine studies found a correlation of *r* = 0.25, closer to that of Smith et al. (2012). This smaller effect size reaffirms the idea that motivational factors are less strongly associated with riot or violent behavior than with institutional or less radical SMs and CB.

**Table 6:** Relationship of affective deprivation and injustice with SM and CB.

**Meta-análisis**	**Factor**	**Years**	***N***	***K***	** $\bar{r}$ **
van Zomeren et al. (2008)	Injustice Affective deprivation	Until 2007	6,344^*^	26	0.49
Smith et al. (2012)	Affective deprivation	1961 to 2010	90,903^*^	157	0.165
Agostini and van Zomeren (2021)	Affective Injustice	2008 up to the present	65,826^*^	207	0.39
				$\bar{\text{r}}$ **=** **0.35**

* Estimated *N*.

Values in bold indicates weighted mean effect size.

Based on *N* = 163,073 subjects and *k* = 390 studies, we find an effect of affective injustice and anger emotion on SMs participation of *r* = 0.35, CI 0.17–0.52, random model.

**Figure 6:**
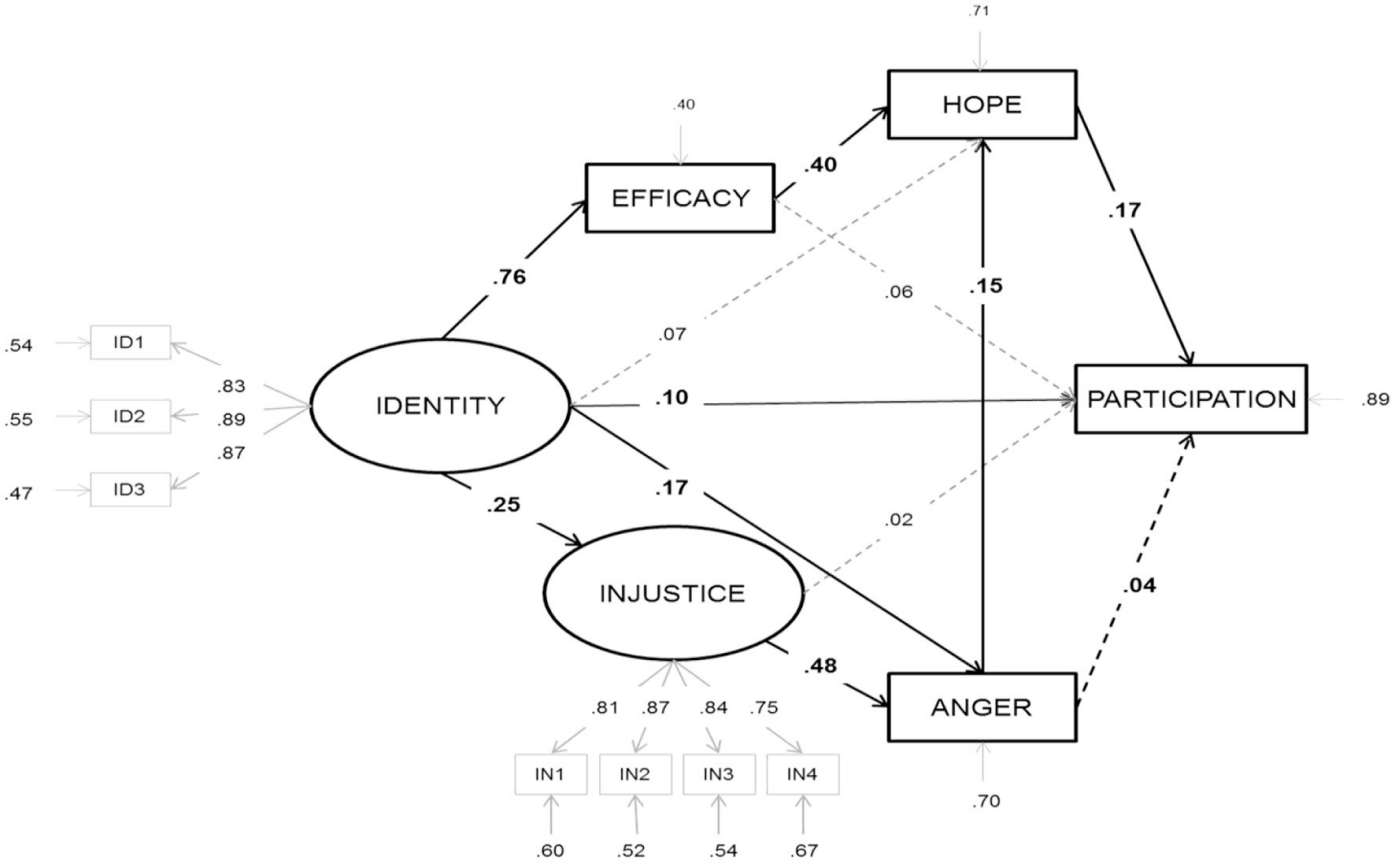
SIMCA model integrating emotions. Source: Wlodarczyk et al. (2017).

Although anger is considered the prototypical protest emotion, other emotions such as contempt, shame, sympathy, and indignation have also been linked to protest. Recent research has also found that hope felt before and pride felt after collective action are important predictors of future participation in collective action (Tausch and Becker, 2013). The emotional climate of SMs is generally characterized by a mixture of festive joy and pride in mobilizing, anger at the injustices being fought, and hope that one can effectively change the situation (Rimé et al., 2017; Sabucedo et al., 2017). Hope transforms a framework of efficacy into actual mobilization. Hope is felt in the face of a negative and uncertain situation, as an alternative positive emotion to sadness and hopelessness. It emerges when one fears the worst but strives for the best or least bad. It is associated with the tendency to feel inspired and to plan for a better future, to be motivated to apply one's skills to the maximum to change negative circumstances. It is the emotion linked to aspiring with some probability that certain desirable group goals will be obtained. Hope can lead to “going beyond the existing”, to building a future that “is not a mere prolongation of the present”. Studies have shown that the emotion of hope is associated with collective efficacy and social mobilization (Sabucedo et al., 2017; Wlodarczyk et al., 2020). The following Figure 6 integrates the emotions of anger and hope into the SIMCA model described above.

### 3.13. Obligation and moral conviction

**Figure 7:**
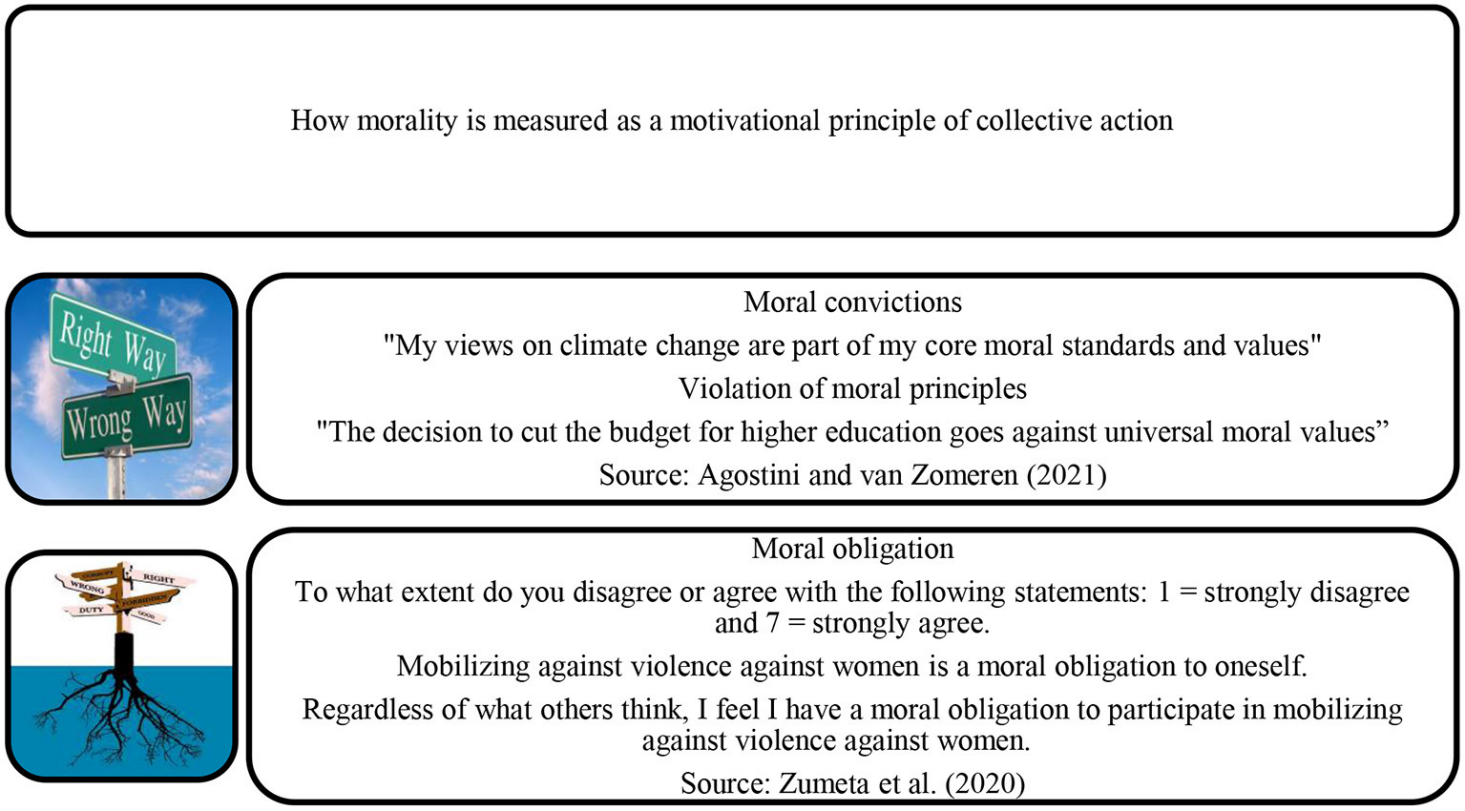
Measures of moral conviction and obligation.

Collective action research has recently emphasized concepts of moral conviction, based on values, moral principles or beliefs, or ideology. If people have moral convictions about an issue (e.g., education as a universal human right) and these convictions are threatened (e.g., when a government proposes to implement tuition fees), then the perceived violation of these moral beliefs and principles, and the resulting emotional experience (e.g., anger), motivates them to engage in action to defend the core values of the attack through collective action (Sabucedo et al., 2018; see Agostini and van Zomeren, 2021). See Figure 7 for operational measurements of moral convictions and obligations.

The meta-analysis by Agostini and van Zomeren (2021) found a correlation between moral conviction or obligation and participation in protests of *r* = 0.36, based on 36 studies and *N* = 24,708. The meta-analysis by Jahnke et al. (2021) examined the relationship between symbolic threat and participation in violent political action among young people. This was defined as the belief that another group poses a threat to the values or views of the ingroup and included concerns about moral beliefs. Based on 10 studies, it found a correlation of *r* = 0.28 between relative deprivation and participation in violent political action—lower but not that different from the previous result.

### 3.14. Ideology: “false consciousness” beliefs and justification of the system

On the other hand, people may share system justification beliefs—that the social system is legitimate and fair, and that differences must be accepted. System justification beliefs, which legitimize disadvantage and encourage acceptance of the status quo, were seen as moral beliefs that hinder collective action (Agostini and van Zomeren, 2021). According to this perspective, people are also motivated to defend, reinforce, and justify the social, economic, and political systems on which they depend. In this sense, it is proposed that there is a general ideological motive to justify the established system or social order, which leads disadvantaged groups to internalize their inferiority, even though this view is detrimental to them. Justification of the system can be explained by epistemic motivation or that people are motivated to justify the status quo since it satisfies the needs for order and predictability. Existential motivation, on the other hand, refers to the fact that many people justify the social system because it helps them to maintain their security needs. Relational motivation refers to people justifying the social system to integrate socially and share a positive view of reality with others (see Marx, 1970; Kay and Jost, 2003; Jost et al., 2017).

**Figure 8:**
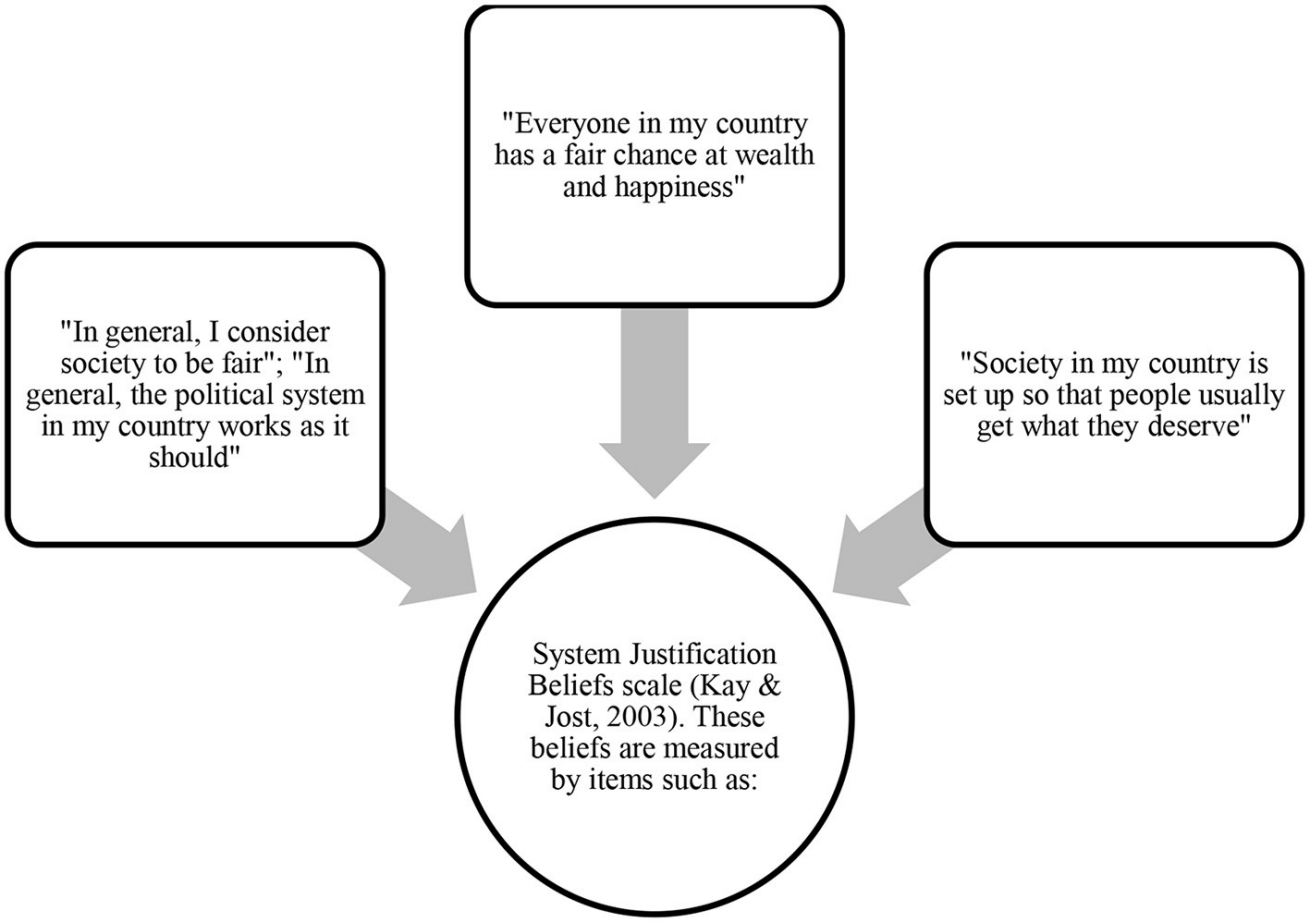
Measures of “false consciousness” ideology.

Agreement with system-justifying or status quo-justifying beliefs (Jost and Hunyady, 2005) would be an ideological factor inhibiting participation in SMs. Although Agostini and van Zomeren (2021) introduced them as moral beliefs, in our understanding, they are ideological beliefs and we have separated them as distinct explanatory dimensions. See Figure 8 for operational measurements of system justification beliefs.

The correlation between system justification beliefs and participation in SMs was *k* = 18, *r* = −0.26—the more people think the system is fair and works well, the less they mobilize. On the other hand, while system justification is negatively associated with collective action that challenges the system, it is positively associated with collective action that supports the system, both for members of low-status and high-status groups (Osborne et al., 2018).

**Figure 9:**
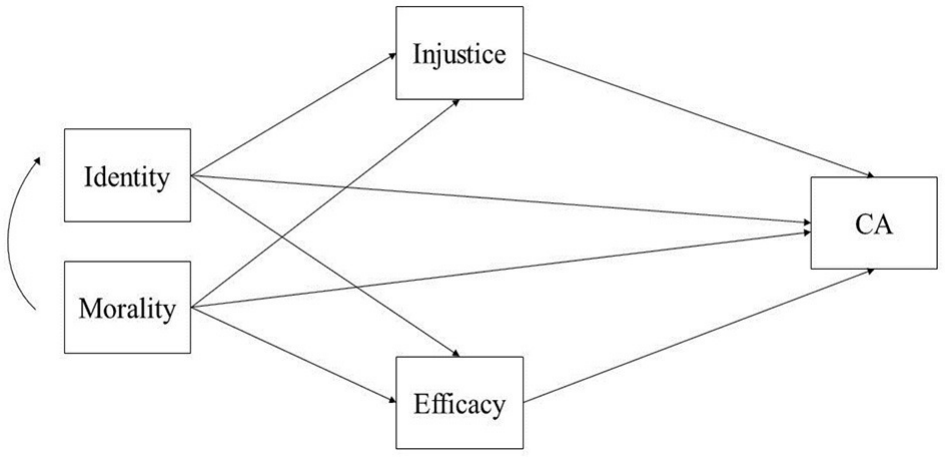
Dual chamber model. Source: Agostini and van Zomeren (2021).

Agostini and van Zomeren (2021) from their meta-analysis developed the “Dual Chamber Model of Collective Action”, which integrates identity, injustice, efficacy, and morality (see Figure 9).

According to this model, morality and identity act as central predictors of collective action, since they in turn activate perceptions of injustice and efficacy. The relationship between both variables is explained because moral convictions define people's social identity, and vice versa; for example, if a person identifies as a regionalist, he or she is likely to defend the interests of his or her community. On the other hand, the Dual Chamber Model explains why members of advantaged groups act in solidarity with the disadvantaged (e.g., men mobilizing for gender equality), to behave congruently with their moral values (Agostini and van Zomeren, 2021).

The following conclusive (Table 7) shows that all factors are strongly associated with participation in movements, showing the greatest strength identity, similar strength efficacy, emotions of affective injustice or RD and morality, and the smallest cognitive injustice.

**Table 7:** Evidence-based explanatory factors for participation in CB and SM.

**Factors SM**	***K***	**Number of meta-analyses**	**Random model effect size**	**Evaluation^a^**
RD/Injustice total	493	3	*r* = 0.30	Medium-strong
RD/Injustice cognitive	216	3	*r* = 0.25	Medium
Efficiency or efficacy	154	2	*r* = 0.36	Strong
Identity	439	3	*r* = 0.41	Very strong
Affective RD as a proxy of emotion anger indignation	390	3	*r* = 0.35	Strong
Morality	77	1	*r* = 0.39	Strong
System justification of the dominant ideology	18	1	*r* = −0.26	Medium

a The mean effect size in social psychology is *r* = 0.24, correlations of 0.30 and above are in the high tactile of effect sizes and above.40 in the highest 25% (Lovakov and Agadullina, 2021).

### 3.15. Cultural differences

Smith et al. (2018) re-analysis used data from their 2012 meta-analysis examining the influence of Hofstede's cultural dimensions on the association between RD and collective action. They used national assessments of individualism-collectivism and power distance to code 303 effect sizes from 31 different countries with 200,578 participants. They found that RD predicted outcomes such as collective action more strongly in individualistic nations. Agostini and van Zomeren (2021) also found that the relationship between identity and participation in SMs was weaker in hierarchical value cultures and stronger in egalitarian value cultures (meta-regression *B* = −0.31 and +0.31). In collectivist cultures, the relationship between identity and SMs was weaker. In western cultures, it was *r* = 0.41 vs. non-western *r* = 0.31. Smith et al. (2018) argue that RD more strongly predicts the outcomes of members of more individualistic cultures in contrast to members of collectivistic cultures because of an internal attribution of causality and greater emotional expressiveness, i.e., because people from individualistic cultures more personally attribute responsibility for their situation and they are more willing to express anger. As a third reason, they argue that individualistic persons perceive their position within social networks and reference groups as less fixed and easier to change. This last explanation seems to us to be the most relevant, and may also explain the greater importance of social identity for participation in CB in individualistic countries. In these cultures, unstable voluntary relationships predominate, which reinforces the importance of affiliation and the emergence of identification in generating social mobilization. In collectivistic cultures, there is a predominance of normative ascribed identities that are stable and make them less mobilizable to motivate protest CB. On the other hand, it is understandable that in cultures of high distance to power, more authoritarian, the relationship between identity and CB is weaker, since these cultures inhibit emotional expression and protest behaviors. However, it can be seen that in most of the relationships between motivational factors and participation in SMs, culture (operationalized as Hofstede's dimensions or Schwartz's societal values) did not moderate these associations.

### 3.16. Theories of the rationality of collective action and collective action frames of SMs

In the case of the explanation of SMs by the rationality of collective action and the creation of “collective action frameworks”, no meta-analyses were found. Therefore, we will summarize the content of the systematic reviews.

#### 3.16.1. Resource mobilization theory and social psychology of expectations and attitudes

Sociologists and political scientists in the 70s and 80s of the 20th century suggested that the availability of resources and the presence of political opportunities as keys to social mobilization—the so-called resource mobilization theory. Groups with more resources and opportunities are more likely to mobilize. Klandermans (1984) developed, from the point of view of social psychology, the Resource Mobilization perspective by applying the Rational Choice attitudinal theory. According to this author, the decision to participate effectively in a CB and SMs is based on a rational choice between costs and benefits, but these go beyond material benefits and costs.

#### 3.16.2. Types of motive for mobilization and empirical evidence

Motives can be divided into three types:

(a) Objective, goals, or collective motives: these refer to the explicit objectives of the mobilization and are also referred to as “collective benefits” since their achievement benefits all members of the social group equally, independent of the commitment that each person has had to the actions of the movement.

(b) Social motives: this refers to the social benefits and costs that the subject obtains by participating. Implies determining the probable reactions of significant others to his or her participation, the importance that the person attaches to these reactions, and the degree to which the subject's network of relationships is involved in the movement—so that participating in it can strengthen this network, becoming an activator of individual behavior.

(c) Reward motives: also called “selective benefits and costs” of the movement, since they only affect those who effectively participate in the mobilizing actions. They involve the costs (time, risk, or financial) and non-social benefits (self-satisfaction and job opportunities, among other possibilities) associated with participating in a given SM.

Empirical studies have tended to show clearly that social motives and goal motives are predictive of participatory behavior. In contrast, reward motives have a lower predictive level (Valencia, 1990; Asún and Zúñiga, 2013). Klandermans (1984) found in research with Dutch union militants that the collective motive had the strongest relationship with participation, followed by the social motive. Reward motive did not explain participation as they did not believe they would have more personal or individual rewards from participating in the action compared to non-participants. Similarly, a late 20th-century study with Basque youth, comparing subjects who did not participate or only participated in legal demonstrations, with radical nationalist subjects willing to engage in illegal mobilizations, found that those who agreed more strongly with participating in “hard” demonstrations did not differ concerning to the personal reward motive. Participants in radical demonstrations showed a similar perception of the costs or punishments for carrying out extra-institutional actions as non-participants. But they showed higher scores for social and collective motives: they believed that the reaction of their environment would be more positive, they believed they had higher expectations of success, and that more people would participate in nationalist demonstrations, and they valued goals more (Valencia, 1990). Similar results were found in a study with regionalist SMs in Chile, although in this case, the social motive predicted participation better than the collective motive (Asún and Zúñiga, 2013).^3^

#### 3.16.3. Conclusion: centrality of social motives and the relevance of social networks

The results of these three studies suggest that participants in SMs and protest CB are not characterized by expecting to receive specific incentives for them, but by hoping to obtain benefits for the group in general. The fact that one of the variables with the greatest capacity to predict the involvement of subjects in protests and SMs is social motives reaffirms the importance of the social networks in which subjects are immersed to understand how they manage to overcome the barriers that hinder participation in collective actions (Asún and Zúñiga, 2013). For all these reasons, there has been a tendency to assign increasing importance to relational variables or integration in social networks or social capital^4^ to explain social protest, interpreted as “social incentives” to participation (Snow and Oliver, 1995; van Stekelenburg and Klandermans, 2013; van Stekelenburg et al., 2013).

The central limitation of this explanation is the postulate that people decide whether to support an action by recognizing and evaluating the attributes associated with it or with the goals of the group that drives them. This rational-economic logic, the logic of expected value, is of an individual type and serves more for instrumental attitudes (oriented to consumer products or concrete behavior) than for symbolic or value attitudes. And it is these symbolic attitudes that are largely associated with participation in SMs. Finally, people participate in demonstrations, although they do not expect them to achieve their objective (Páez and da Costa, 2022).

#### 3.16.4. Types of collective action frames

The collective action frameworks approach explained mobilization as a three-step process involving: (a) diagnostic framing: where the leaders who generate the SMs construct their interpretation (diagnosis) of what appears to be the problem that requires remedy, as well as the attribution of blame to the culprits (individuals, groups, and institutions); (b) prognostic framing: where a way out of the problem is suggested, a viable solution to alleviate or eliminate the undesirable situation being experienced; (c) motivational framing: where a rationale for action is provided to explain why mobilization by constituents is necessary, why it makes sense to react, and how engagement in collective action may be the answer to shared problems (Snow and Oliver, 1995; Benford and Snow, 2000). Gamson (1992) posited three alternative collective action frames: injustice, identity, and agency. The injustice frame is considered by many scholars to be crucial in the formation of collective action and all successful movements employ an injustice frame. However, Snow and Oliver (1995) successfully refuted this claim by citing religious, self-help, and other SMs that do not use such a frame.

#### 3.16.5. Collective action frames, motivational factors of SMs, and absence of common beliefs

As can be seen, the frames of injustice, identity, and agency are similar to the motivational factors already reviewed—agency refers to collective efficacy–or to the principles of Touraine's SMs—diagnosis equals identity and opposition and prognosis equals the totality or project of the SMs (Páez and da Costa, 2022). Furthermore, this approach shares the idea that there is a set of common beliefs that explain participation in CB and SMs, an idea that had already been questioned in relation to CB studies that appealed to generalized beliefs and emergent norms.

Frame analysis is a content analysis of SM discourses, and the process of identifying a frame is itself a process of subjective interpretation. A large number of frames have been described; the studies tend to be descriptive and we believe that in reality, this approach only adds to take into account the social representations of SMs—it remains to be ascertained which and to what degree they are shared by the participants in them. At odds with this approach, it was found that having a favorable attitude toward an SM objective did not imply actual participation in CB and that a part of the protesters did not identify with the organizers of these (van Stekelenburg et al., 2013).

#### 3.16.6. Conclusion: limitations of collective action frames as explanations of SMs

As Johnston (2003, p. 209) put it:

“Currently framing analysis is in crisis...Recently researchers have shown concern regarding several trends, the most important of which is a preference for descriptive research rather than a causal view of frames. In other words, framing research has so far been very effective in describing the complexity of the ideas involved in mobilizing people, but not in testing propositions about the mobilizing power of frames.”

### 3.17. Effects of participation in SMs and CB in the medium and long term

Emotional, identity, social integration, empowerment, and ideological and knowledge effects of participation in SMs are described. They are based on the systematic review of Vestergren et al. (2017) of 57 studies on biographical consequences of participation in SMs, Ni et al. (2021) systematic review of 52 studies on mental health during and after CB and SMs, as well as studies on the first topic, realized after the 2017 revision.

a. As for emotional effects, it has been found that participation in SMs influences emotional responses and future action intentions. Tausch and Becker (2012) found an increase in pride after CB success and that this emotion influenced action intentions, through increased perceptions of efficacy (one study, Vestergren et al., 2017). A longitudinal study found that participation in anti-terrorism and anti-war demonstrations in a relatively successful context predicted more positive emotions and a more positive emotional climate 3 months later, although it also maintained negative emotions linked to the attack (Rimé et al., 2004). On the other hand, a Swiss longitudinal study using panel data found that intention to participate in demonstrations slightly predicted negative emotions, and intention to participate in strikes decreased positive emotions, as well as that emotional distress predicted intention to participate in CB (Lindholm, 2020). Cross-sectional, 3-month and 1-year follow-up studies have found that participating in unsuccessful SM demonstrations (such as the Umbrella movement in Hong Kong) while inducing positive emotions at the climatic moment of the CBs, causes a decrease in these when the movement fails (Hou and Bonano, 2018; Chan et al., 2021; Fung, 2022). These results suggest that participation in SMs, particularly unsuccessful ones, has an emotional cost, although in the case of successful ones, this is compensated by improvements in the emotional balance.

b. Effects on identity and social relations of participation are very important, highlighting long-term commitment to the group, such as sustained participation in SMs (confirmed in 18 studies by Vestergren et al., 2017), as well as extended participation in other causes and struggles (confirmed in seven studies by Vestergren et al., 2017). Participation strengthens collective identity, which in turn affected participants' sense of personal identity (confirmed in three studies by Vestergren et al., 2017). Both the formation of new very strong and close relationships and the effort of participation in the SMs cause stress and strain that affect personal relationships (confirmed in three studies by Vestergren et al., 2017).

c. Significant empowering effects have also been found, such as increasing our beliefs that we can collectively achieve something and increasing self-confidence (confirmed in six studies), increasing participants' level of self-esteem (in three studies), improving wellbeing (in three studies), and increasing the belief that the world is changing (eight studies—all in Vestergren et al., 2017).

However, the other side of the coin, disempowerment from SMs defeat (three studies) and also burnout or exhaustion (three studies, in Vestergren et al., 2017), has also been found. One longitudinal study found that youth with high online and offline participation in the Umbrella SM 2014 in Hong Kong showed significantly higher levels of psychological and social wellbeing, higher leadership competence and political control, as well as lower perceptions of government responsiveness during the period of street occupation. In the year after the movement, youth with high participation had a significant decline in psychological and social wellbeing compared to other youth groups (Chan et al., 2021). Panel studies between 2009 and 2020 found a negative impact of CBs on the mental health of the general population: more potential depression during 2019–20 (11–12% respondents with high symptoms of depression) and 2017 (6–5% mobilizations compared to previous years; 1–9% during 2009–14). Participation in demonstrations was not associated with depression, although it was associated with PTSD symptoms (Ni et al., 2020) probably because of exposure to repression and violence. A review of 52 studies confirmed the negative impact of CBs. It was found that after a major protest, the prevalence of probable major depression increased by 7%, regardless of personal participation in protests, as a general effect in the community. Six longitudinal studies support this finding. Factors associated with depressive symptoms included exposure to violence, interpersonal conflict, frequent use of social media, and lower social support. However, two studies of ethnic riots in the US and Northern Ireland suggested that collective actions may reduce depression, possibly because collective actions serve as a positive experience when people collectively voice their grievances. In addition, greater social cohesion among subpopulations, which support or oppose the cause of collective action, could strengthen social ties, which in turn could buffer the adverse impact of the stressful environment and greater social cohesion within subpopulations of rioters (Ni et al., 2020).

d. Effects or changes in values, beliefs, ideology, and knowledge: participating in SMs has been associated with ideological radicalization (confirmed in 18 studies), learning new skills (in four studies), and new knowledge (in four studies, all by Vestergren et al., 2017).

It has been criticized that many of these results are based on movements of the New Left of the 1960s in the USA and have limited external validity. However, a longitudinal study in Switzerland showed that previous participation in peaceful mass demonstrations in 1999 influenced people after 15 years: participants in 2013 reported more left-wing attitudes, voting left, and remaining linked to SMs and partisan organizations (Giugni and Grasso, 2016).

## 4. Discussion

The review confirmed that participation in collective meetings is high, particularly in non-ideological leisure CBs, while participation in religious CBs and demonstrations and in ideological CBs linked to SMs is a minority.

When asked why people mobilize, as we have seen, there are different partial explanations, which are partly supported by studies on the characteristics and patterns of CB, as well as by Pizarro et al. (2022) meta-analysis on the effects of participation on CB and feelings of collective effervescence:

a) People mobilize because they are aggrieved as groups, particularly if this upsets them emotionally, and provokes indignation; however, people participate in SMs, although its ingroup does not suffer deprivation. On the other hand, religious, self-help, and other non-contentious SMs movements do not rely on deprivation and injustice (Snow and Oliver, 1995). Congruently, participation in CB is related to specific moments of intense emotionality, and protest CB or demonstrations increase negative emotions—but religious and positive valence CB are not related to negative emotions (Pizarro et al., 2022).
b) People mobilize because they have the resources and perceive that there are opportunities. Subjectively, people mobilize because they value the collective objective and expect the behavior to succeed, because they expect collective and social benefits to be obtained (their environment will approve of them)—although having a favorable attitude toward the objective of the movement is not enough to mobilize. The absence of blind violence in riots and functional CB or resilience in catastrophes supports explanations of SMs by the rationality of collective action.
c) People mobilize because they believe they are effective or capable of controlling the environment and distrust institutions. Participation in CB such as riots is related to political efficacy (Allen, 1970), and participation in protest CB is related to collective efficacy (Pizarro et al., 2022).
d) People mobilize because they share and create a social identity oriented toward political action. Participation in protest CB is related to collective identity or social identification and fusion with the group of demonstrators (Pizarro et al., 2022). However, some people participate in demonstrations without sharing identification with leaders or without reporting any identification at all.
e) People mobilize because they share the view of their group's situation as unjust and feel anger, as well as that their group is effective and feels hope. They also perceive that anger and hope are shared by people in their social group—they perceive an emotional climate favorable to mobilize. Participation in protest CB is related to personal and collective positive emotions, such as hope or social awe, as well as negative emotions like anger (Pizarro et al., 2022). People mobilize because they feel moral outrage and feel they have a moral obligation to mobilize. CB studies suggest that extreme demonstrations such as ethnic riots and lynching follow a “moral logic”.
f) People mobilize because they challenge the dominant ideology and social system—in the case of contentious SMs who want to change the social order. System justification beliefs and related social beliefs (RWA and SDO) sustain reactionary or conservative SMs. However, studies on demonstrations showed that people did not share homogeneous beliefs about the situation and objectives of the mobilization, partially questioning collective action frameworks but also ideological explanations of SMs. Repeated participation in CB reinforces the agreement and convergence of opinions on the SM social representations or narratives that integrate frames of identity, opposition or conflict, injustice and anger, and effectiveness and hope.

Although there are no meta-analyses on the topic, we showed that the medium- and long-term effects of participation in SMs and CB lead to positive emotions, psychological wellbeing, general social identification, and empowerment or increase in self-esteem and self-efficacy (Pizarro et al., 2022).

However, periods of mobilization seem to have a negative impact on the wellbeing of the population, although not necessarily on the protesters. The defeat or setbacks of mass movements are associated with disempowerment (lower wellbeing and burnout). In the case of SMs that have not suffered major defeats, even if they have not been very successful, participation has a persistent impact on ideology.

### 4.1. Limitation of review

There are limitations to the social psychology studies of SMs reviewed. The first limitation is that mainstream social psychological models are weak. The mainstream social psychological models are of functional relationships and there is no strong theoretical argumentation behind them. The SIMCA and ESIMCA models are based on the correlations found and differ in the order of the arrows and the last dual model is a variation of the previous ones. Empirical tests show that different interrelationships fit the data and not only those postulated by the models (see, for instance, Zamudio et al., 2022).

A second limitation is that sometimes the variables (e.g., mobilized or politicized identity) that predict collective action are so close or similar to those they explain that they can be quasi-tautologous, e.g., I identify with mobilized students and I mobilize.

A third limitation is that studies do not differentiate between long-term CB or engagement with SMs and one-off participation, at least with sufficient finesse. It is necessary to conduct longitudinal and mixed studies in a dialogue among survey, experiment, quantitative, and qualitative observations. Likewise, it is necessary to examine the sequences of manifestations and their effects on the maintenance/decrease of SMs.

A fourth limitation is that the difference between individual (personal and group) and collective emotions is not taken into account (see Pizarro et al., 2022 for this distinction). Chronically felt emotions shared by people, based on cultural settings, values, and norms can form a collective mood and emotions, an emotional atmosphere and climate. The initial emotional climate of SMs is generally characterized by a mixture of festive joy and pride in mobilizing, anger at the injustices being fought, and the hope of being able to effectively change the situation—as the analysis of the evolution of the emotional climate in the Tunisian Arab Spring showed (Rimé et al., 2017). Participation in demonstrations is associated with a perception of a negative emotional climate, where collective anger is perceived. Participants in the 15-M mobilization in Spain in 2011 perceived more social injustice, consistently shared more strongly a perception that anger and hostility predominated in society, and also perceived a lower positive emotional climate. This was explained by the minority and resource-limited nature of that movement. However, participants in a Catalan nationalist demonstration (the 2013 Diada), although they also perceived more injustice than non-demonstrators and a higher level of negative collective emotions, perceived a greater positive climate than non-participants. Non-participants disagreed that there was a situation of injustice, although they also perceived that there was greater hostility, less joy, and tranquility to speak. The greater resources and expectations of success in that period were reflected in the nationalists perceiving a better emotional climate (Sabucedo et al., 2017). On the other hand, mobilizations cause changes in emotional climate: participation in protest demonstrations against the Atocha bombings predicted a better emotional climate 2 months later. Finally, the emotional climate acts as a context and influences people: the perception of a positive emotional climate 1 week later, i.e., that the majority was supportive after the Atocha bombings, predicted greater positive affectivity and social support after 3 weeks (Páez et al., 2007). Perceiving supportive collective behavior acted as a resilience mechanism, which helped to recover wellbeing (Sabucedo et al., 2017).

The fifth limitation is that there are external validity issues in the studies reviewed. Many of the samples are western and student samples and studies from other cultural areas are missing—although in the reviews by Agostini and van Zomeren (2021), as well as Smith et al. (2018), there were studies in collectivist and high-power distance cultures, and the moderating effect of culture was contrasted. It was found that in individualistic cultures, the relationship among RD, identity, and participation in SMs is stronger than in collectivistic cultures, probably because of the greater attribution of internal responsibility to the individual, the greater acceptance of the expression of emotions, and because collective identities are less fixed and more mobilizable in these cultures than in collectivistic ones.

Another limitation of the literature reviewed is that the role of intergroup conflict or instrumental threats, resource contention, and social conflict are not developed. We believe they play an important role in contentious SMs, although see Jahnke et al. (2021) analysis of exposure to intergroup conflict and instrumental or realistic threat,^5^ as factors facilitating participation in violent CB. In the same vein, of the need to take into account aspects of the social structure, it has been found that intergroup contact between social categories of different statuses plays a role in SMs. Positive intergroup contact between high- and low-status categories provokes rejection of participation in SMs among disadvantaged group members. Positive contact with higher status groups can feed the illusion of individual mobility and the belief in a common social identity, as well as reinforce the justification beliefs of the system, acting as a “sedative or opium for disadvantaged people”. At odds, positive intergroup contact is associated with increased support for progressive SMs or social change toward equality among advantaged group members. In the last case, positive attitudes toward disadvantaged group members may increase individuals' engagement in solidarity-based collective action, and positive intergroup contact fuels participation in progressive SMs (Cakal et al., 2011; Hässler et al., 2020).

As a future element, we believe it is necessary to integrate repeated measurements of the ideologies, frames, and social representations of the SMs, as these explain in part their social base and dynamics. Even if the same explanatory principles serve for Black Lives Matter or the Proud Boys and Trumpist groups, the content and degree of impregnation of their social beliefs are relevant. Concerning CBs, particularly linked to secular and religious parades, celebrations, and rituals, other motivations appear important. A meta-analysis of motives for participating in collective leisure and recreational gatherings found a large number of motivations different from those analyzed in CB and SMs related to self-improvement, personal growth, learning, creativity, stimulation, and autonomy—in addition to affiliation (Manfredo et al., 1996; Cheng and Pan, 2012).^6^ It can be seen that, despite the rather descriptive nature of this type of literature, other motivations, in addition to injustice and moral and ideological beliefs, can explain participation in positive, playful, and non-contentious CB and SMs. It appears that collective identity and positive emotions play a role and that motivation of affiliation, achievement, development, and creativity, as well as stimulation and repair, are important (Castro-Abril et al., 2021). A meta-analysis found that the frequency and diversity of participation in leisure activities, such as social activities, sports, games, and cultural experiences, which tend to be generally performed in collective gatherings, was related to subjective wellbeing *r* = 0.26 (Kuykendall et al., 2015). In this sense, we believe that the study of CBs linked to parades and celebrations is an area of study to be developed. Conclusions about the long-term positive effects of participation in CBs and SMs are limited by the absence of a meta-analysis, which is a pending task for future. Long-term conclusions are also limited because studies usually evaluate retrospectively or only weeks or months after CBs and SMs' psychological changes and many are based on comparisons between activists or participants and non-participants, although some studies are long-term longitudinal. Finally, most samples are western ones, which limits the cross-cultural validity of the results.

## 5. Final conclusion

In conclusion, participation in collective gatherings is frequent, mostly of a leisure type, to a lesser extent religious and sporting, and even lesser extent demonstrations and large religious rites. Although 50% of people report they do not participate in demonstrations, these are frequent and four out of 10 CB protests and SMs have some degree of success. It is necessary to expand the studies of positive valence CB since these are scarce.

Meta-analysis shows that collective identity is the most important psychosocial factor of SMs, particularly emergent politicized identity. However, a significant group of participants did not identify with the objectives of the movement and a relevant minority did not identify with collective motives or participants. This shows the complexity of social identification and questions the general explanatory role of collective identity in SMs.

The second psychosocial factor of participation in SMs was moral conviction. The importance of moral beliefs as justification for participating in CB was also confirmed. Collective efficacy is a third important explanatory factor of SMs. Affective relative deprivation related to negative emotions, such as anger or moral rage, and cognitive relative deprivation or injustice are the fourth and fifth factors that explain protest CB and SMs. However, they do not play a role in non-contentious CB and SMs. The role of positive emotions should be further explored in the future—not only in positive valence CB but also in conflictual SMs, because they seem to play an important role. The sixth and last factor of SMs is disagreement with system justification beliefs, suggesting the importance of ideology and shared beliefs. However, participants in demonstrations did not share homogeneous beliefs about the situation and objectives of the mobilization, partially questioning these explanations of SMs. Probably shared beliefs, or the agreement with the collective action ideological frameworks, are generated and generalized as an effect of participation in SMs—rather than being a prerequisite. The study of social representations, linked to SMs and CB, their content, the level at which they are shared, and how they evolve, is an area that deserves to be further developed.

We believe that longitudinal studies that combine push factors before participation in CB and the processes during these, to explain the outcomes in the medium-term and the long-term dynamics of SMs, are necessary. As an instrument to help this, Appendix 2 contains a protocol for assessing CB and participation in SMs, which is based on Wlodarczyk et al. (2020), Zumeta et al. (2020), Castro-Abril et al. (2021), and Pizarro et al. (2022) in this issue and all subsequent previous and ongoing studies developed by the Consolidated Research Group on Culture, Cognition, and Emotion and its external collaborators. It is hoped that it will be useful for the development of new research to accumulate evidence-based information for future meta-analyses, as well as to inspire new studies in this area, such as the line of research mentioned in the protocol.^7^

## Author contributions

SdC and DP conceptualized and organized the present study and prepared the first draft. VD and MM-G collaborated in the coding of the studies. Meta-analytic integration was performed by DP and SdC using CMA 3.0. VD and PB were responsible for the translation and revision of the text. All authors reviewed and contributed to the different versions of this article, and the improvements made in the latest versions of PB were also relevant. All authors contributed to the article and approved the version presented.

## Footnotes

^1^The definition of CB as a coordinated interaction of a group of people in a collective gathering seems valid to us. This does not exclude that a protest CB can be carried out by members of a group in favor of disadvantaged outgroups (men who support the feminist movement, or whites who mobilize against anti-black racism). But McPhail and Wohlstein's definition refers to what happens in the micro context of a demonstration.

^2^Data obtained from https://www.worldvaluessurvey.org/WVSOnline.jsp.

^3^Operationalization of collective motives or goal expected value: (a) Value of collective good: participant in SM were asked how they stood for the goal or how important was the goal—on a 5-point scale from very positive to very negative or not at all important = 1 to very important = 7. (b) Expectation that participation helps to achieve the collective good: “In your estimate, how many people will participate in the collective action?” (very few, not so many, quite a few, very many), “Imagine that very many people take part in the collective action. Do you think that this will induce (Government, employers…) to accept what is demanded or to take into account our claims?” (Klandermans, 1984; Valencia, 1990). A meta-analysis was carried out with the data of Klandermans (1984), *r* (565) = 0.26, Valencia [1990, *r* (146) = 0.60] and two studies asking for agreement with SM goals or values [Castro, *r* (252) = 0.56; Páez et al., 2013, *r* (246) = 0.29]. Based on *N* = 1,209 and *k* = 4 studies, a strong effect of attitudes or expectations as collective motive on participation in SM was found—random model *r* = 0.44, CI 0.25–0.49. Using only the data of two first studies specifically applying the expected value model, the effect size found was similar, *r* = 0.44.

^4^No meta-analysis was found for social capital and participation in CB or SM.

^5^They define exposure to intergroup conflict as witnessing ingroup members fight or get wounded in a confrontation with the outgroup (directly or indirectly through media reports) and realistic threat as the belief that another group threatens the ingroup's welfare (e.g., through competition over power or resources). They found a strong association between exposure to conflict and realistic threat with disposition to participate in violent political actions or CB, *r* = 0.24 based on seven studies and *r* =0.30 based on 50 studies, respectively.

^6^A meta-analysis of reasons for participating in collective leisure and recreational gatherings found 39 important descriptive motives (Manfredo et al., 1996; Cheng and Pan, 2012). Following psychological needs studies (Sheldon et al., 2001), they were reorganized into 15 motivations. (1) *Stimulation*: novelty, obtaining excitement and stimulation; (2) *Hedonic:* Have fun, occupy my free time; (3) *Self-esteem:* social recognition, creating a good impression in front of other people; improving their self-esteem; (4) *Relatedness or Affiliation:* Do things with family and acquaintances; be with like-minded people; being with friends; meeting friends again; being with people who do the same things and have the same values; meeting and being with new people; observing and meeting new friends; meeting people; social sharing (Tell other people you were in the activity); (5) *Competence:* developing skills; evaluating their competences; reinforcing self-image, increasing self-confidence, pride and competence; (6) *Power*: experiencing leadership; be able to control things that happen; (7) *Autonomy*: satisfy need for independence; autonomy freedom make your decisions; (8) *Creative*: creativity, be creative do creative things; live new experiences; (9) *Self-actualization*: develop spiritual values grow spiritually; think reflect on themselves; (10) *Learning:* learning in general, expanding my education; knowing the geography and terrain; learning, enjoying more about nature and the landscape; (11) *Express collective identity*: nostalgia remember things past or relive the history of the community; (12) *Thriving:* improve physical fitness (exercise and be active); (13) *Security*: social security, to be near considerate people, risk reduction and avoidance; (14) *Aesthetic*: enjoy scenery, nature; (15) *Affect regulation*: rest relax physical; escape social pressures; recover from stress; calm down mentally; escape from overloaded work roles and get away from the demands of daily life. Collective identity (for instance, motivations 3, 4, and 11), self-efficacy (see motivations 5, 6, and 7), positive emotions (see motivations 1 and 2), moral convictions, and ideology (see motive 9) are factors that could be related to some of these motivations. But, it is not the case of creative, learning, esthetic, thriving, affect regulation, and security motivations, that looks specific to positive valence CB. Moreover, factors such as perceived conflict, threat, RD cognitive, and affective (anger and moral outrage) did not play a role in positive valence CB.

^7^See the project at osf.io/ec6fb.
