# Supplementary material for: Social movements and collective behavior: an integration of meta-analysis and systematic review of social psychology studies
Source: Front Psychol. 2023 Apr 21;14:1096877. doi: 10.3389/fpsyg.2023.1096877 (PMC10162496; doi:10.3389/fpsyg.2023.1096877)
Supplement: Supplementary file 1 [file Data_Sheet_1.PDF]

# Complementary material 1

| Glossary of terms | |
|---|---|
| **Collective Action** | Collective action can be conceived from a macrosocial perspective as a collective challenge to the socio-political system, closely linked to social movements. From a more microsocial or psychological perspective, collective action refers to action taken by a group of people in response to social issues to benefit a group or society as a whole (Bou Zeineddline & Leach, 2021). Collective action is also defined as behaviors enacted with the goal of ameliorating perceived disadvantages faced by one's psychological in group, but also as behaviors carried out by members of groups not directly targeted by a perceived injustice, due to feelings of psychological closeness and identification with the disadvantaged group (Craig et al, 2020) |
| **Collective Behaviour** | Two or more interacting individuals engaged in one or more behaviours (e.g., orientation, locomotion, gesticulation, tactile manipulation, and/or vocalization) that can be considered common or convergent along one or more dimensions (e.g., direction, velocity, tempo, and/or substantive content) at the same time and that involve coordination (McPhail & Wohlstein, 1993; Snow & Oliver, 1995) |
| **Collective Gathering** | Meeting of two or more people who interact with each other over a period of time for a given purpose (e.g., union work meeting) (Páez & da Costa, 2022; Collins, 2009). |
| **Collective Efficacy** | Refers to the individual's expectation that it is possible to change conditions through collective action. For the perception of the possibility of change to take hold, people must perceive that the group is capable of uniting and fighting for the issue, and they must perceive that the political context is receptive to their group's demands. The first refers to group efficacy: the belief that group-related problems can be solved through collective efforts, and the second refers to political efficacy: the belief that political actions can impact the political process (van Stekelenburg et al., 2013; Snow & Oliver, 1995). |
| **Collective Identification** | Subjective degree of affiliation or belonging to a collective or social group. A shared definition of a group that derives from the common interest and solidarity of its members (Snow & Oliver, 1995). The more people identify with a group, the more likely they are to protest on behalf of that group (Jahnke et al., 2021). |
| **Crowd** | In crowds, people gather -face to face, acting spontaneously- around a common object of attention or purpose, such as the expression of a labour protest in front of a public building (Snow |

| | |
|---|---|
| | & Oliver, 1995). |
| **Demonstrations** | If collective encounters facilitate collective protest behaviour, it is a demonstration generally framed within a social movement. Demonstrations or protests are forms of collective action related to social movements that are defined as any temporary occupation by several people of an open space, public or private, that directly or indirectly includes the expression of socio-political opinions (Filleule, 1997; Snow & Oliver, 1995). |
| **Emotions** | Can be distinguished from mere feelings or moods by their relation to a specific object or idea. Emotions of collective action are felt towards the opponent, the in-group, and contentious issues. Negative intergroup emotions are the experience of negative emotions on the ingroup's behalf. Protesters are likely to experience negative emotions towards their opponent(s) and the contentious issue that motivates collective behaviour, while they most likely feel positive emotions towards the group(s) they identify with increasing commitment to SM. Perceived **collective mood or emotional climate** resulting from group membership and experience provides feedback to people about how the group (namely, the political community) is doing. Research has demonstrated that people in a positive mood display more self-efficacy, are more optimistic, and show more associative cognitive processes, while a perception of negative emotional climate fuels CB (de Rivera & Páez, 2007; Jahnke et al., 2021; van Stekelenburg, 2013; Snow& Oliver, 1995) |
| **Moral obligation** | It has been defined as the belief that one should act according to their own values and principles (Sabucedo et al., 2018). |
| **Moral or symbolic threat** | Belief that another group poses a threat to the values or views of the ingroup; may include concerns about moral beliefs (Agostini & van Zomeren, 2021; Jahnke et al., 2021) |
| **Parade** | If the collective gathering facilitates expressive behaviour or is an expressive collective ritual, party or celebration, it is a parade (McPhail & Wohlstein, 1995). |
| **Political Action** | Political action is any form of organized political act carried about by a group of people to attain a purpose by activity in political channels. Collective political actions are challenges by people with common purposes and solidarity in interaction with elites and authorities. Collective political actions are related to social issues and conflicts, and concern people with a common purpose and solidarity (Klandermans, 2015) |
| **Relative Group Deprivation** | Belief that someone's group is worse off compared to other groups in society (Smith et al., 2012) |
| **Riot** | "Riots" are collective encounters involving crowds committing |

| | |
|---|---|
| | individual or collective violence against people or property - such as hooliganism at sporting events or ethnic and racial riots (McPhail & Wohlstein, 1983; Páez & da Costa, 2022). |
| **Realistic threat** | Belief that another group threatens the ingroup's welfare (e.g., through competition over power or resources (Jahnke et al., 2021) |
| **SM or mass movements of long duration** | They are collective challenges based on common objectives and social solidarity in a sustained interaction with elites, opponents and authorities (Tarrow, 1997). They include regular collective behaviors of protest and celebration. Protest, marches, rallies, celebrations are often associated with social movements (Snow & Oliver, 1995). |
| **Social networks** | Individual grievances and feelings are transformed into collective grievances and feelings within the group. Networks function as a "socialization device" and a "recruitment device". Individuals feel more likely to participate in a protest as long as they are embedded in social networks, which provide opportunities to discuss and learn about politics (van Stekelenburg et al., 2013; Snow & Oliver, 1995). |
| **System justification beliefs** | Beliefs that society is just and is as it should be, thus supporting the maintenance of the status quo. This justifying "false consciousness" of society permeates even subordinate groups, as it assures them of cognitive stability (Jost et al., 2017) |

Bibliography[1]

Collins, R. (2009). *Cadenas de rituales de interacción*. Barcelona: Anthropos.

Craig, M. A., Badaan, V., & Brown, R. M. (2020). Acting for whom, against what? Group membership and multiple paths to engagement in social change. *Current Opinion in Psychology, 35,* 41-48.

de Rivera, J., & Páez, D. (2007). Clima Emocional, Seguridad Humana y Culturas de Paz. *Revista de Asuntos Sociales, 63* (2), 233–253. https://doi.org/10.1111/j.1540-4560.2007.00506.x

Klandermans, B. (2015). Collective Action. In J.D. Wright (Ed.). *International Encyclopedia of the Social & Behavioural Sciences* (Second Edition), (pp. 145-150). Oxford: Elsevier.

[1] References not listed in the Bibliography of the article are added here.
