# Supplementary material for: Social movements and collective behavior: an integration of meta-analysis and systematic review of social psychology studies
Source: Front Psychol. 2023 Apr 21;14:1096877. doi: 10.3389/fpsyg.2023.1096877 (PMC10162496; doi:10.3389/fpsyg.2023.1096877)
Supplement: Supplementary file 2 [file Data_Sheet_2.pdf]

Complementary material 2

PROTOCOL ON SOCIAL MOVEMENTS AND COLLECTIVE BEHAVIOUR

Culture, Cognition and Emotion Research Group (CCERG)[1] and external collaborators involved in the research of social movements and collective behaviours [2]

Abstract

This protocol operationalizes the predictor variables, the frequency of CB and SM participation, the processes at work during CB episodes, and their outcomes.

A first set of variables and instruments aims at examing the level of participation and the factors that lead to participation in SM. Variables of psychological well-being and emotional climate are described as possible criteria for assessing medium-term effects.

A second set of variables and instruments can be used to analyse the processes that occur in collective encounters or gatherings (CG) and behaviour (CB), such as demonstrations, collective rituals, meetings, celebrations linked or not to social movements (SM), and their immediate effects.

This protocol, based on published and reliable open-access instruments, allows for the investigation of CB and SM, adapting the content to their type and subject matter.

Introduction

This text proposes a minimum common protocol for SM and CB based on previous work (see literature review section) and the theoretical review carried out in this article. However, it is necessary to take into account a) the similarities and differences between both constructs, as well as b) the level of analysis at which each of them is located: macro level in social movements and more micro level in collective behaviour.

Justification

The frequency and repercussion of SM and CB associated (or not) with them, support the importance of approaching this phenomenon from a social psychological perspective. Before and during the period of the Covid-19 pandemic, collective outbursts of protest and large mobilizations have shaken Chile, Colombia and other countries. In recent decades, collective behaviour of protest and SM have occurred in more than 180 countries, including 99% of the world's population. It is estimated that the nearly 5,000 revolts that have occurred in 158 countries during Covid-19 have demanded recognition

[1] https://www.ehu.eus/es/web/psicologiasocialcce

[2] This opens the research line: "Social Participation, Adaptation to Change, Health and Well-Being in Groups and Organizations: A Gender-Sensitive Project" (da Costa et al., 2021).

of economic and social rights, in addition to showing the underlying vulnerability and social inequities, having a negative economic impact of losses of 15 billion dollars. This project seeks to empirically corroborate the motivational factors that explain participation in SM and CB, as well as the medium and long-term psychological effects of participating in them.

Comparative characteristics of SM and CB

Definition and characteristics	SM	CB
Collective or group in which there is interaction	X collective or group	X group
They are informal	X	XX
They act with continuity	X to a large extent	
Sense of unity or collective identity	XX	X
Institutional common action	X	
Extra-institutional common action	X	
Are collective challenges	X	X some
Are based on common and clear objectives	X	X sometimes
Based on social solidarity	X	X sometimes
Sustained interaction with elites, opponents and authorities	X	In general, no
Emerge as a collective response to the malaise created by a social problem or around a social conflict	X	X but on a one-time basis
Level of continuity (high)	X	it depends
Organized	X	X
Promote or resist changes in the society of which they are a part or beyond it	X	X but on a one-time basis
Pursue a political agenda or common cause	X	to some extent
Use a variety of strategies to achieve their objectives	X	
Use interpretive frameworks to define a problematic situation, raise awareness and motivate others to gain their support	X	
Express identities, grievances and social goals	XX	but on a one-time basis

Bibliographic review

As a complement of the articule, this section presents previous research and projects on this line of work, carried out within the framework of the CCERG and its external collaborators. Relevance is given to articles published in the last 10 years, since they include previous advances on this line of research.

Authors	Year	Type of publication	Quality	Contents
Rimé, B. & Páez, D. https://www.doi.org/10.1177/17456916221146388	2023	Perspectives on Psychological Science	Peer Review	Examines Durkheim model of collective gatherings, discussing current theories, and evidence, that support and nuance it. Propose a global model of CG framing CB protocol
Cusi et al. https://e-revistas.uca.edu.ar/index.php/RPSI/article/viewFile/4237/4206	2022	Revista de Psicología	Peer Review	The frequency of participation in collective meetings is associated with collective effervescence and through these to well-being and emotions of transcendence.
Castro et al. https://www.doi.org/10.3389/fpsyg.2021.764434	2021	Frontiers in Psychology	Peer Review	Identification with the protesters, agreement with their grievances, high perceived emotional synchrony, and higher creativity responses were associated with active participation in the social movement
Bouchat et al. https://www.doi.org.10.1111/jasp.12649	2020	Journal of Applied Social Psychology	Peer Review	Participation in a collective gatherings (major scouting event) provokes positive effects
Zumeta et al. https://www.doi.org/10.3389/fpsyg.2020.607538	2020	Frontiers in Psychology	Peer Review	Collective participation brings positive individual and collective benefits, reinforced

				through psychological mechanisms such as collective effervescence, identification with the group, in line with the Durkheimian approach to collective rituals.
Wlodarczyk et al. https://www.doi.org/10.3389/fpsyg.2020.01721	2020	Frontiers in Psychology	Peer Review	Proposed an integrative definition of PES. Structural validity of the original PES scale. Incremental validity of PES is examined in two longitudinal studies, particularly with respect to well-being. Proposed an integrative short form of the PES Scale, which measures antecedents and behavioral effects of collective effervescence
Páez et al. https://www.doi.org/10.1037/pspi0000014	2015	Journal of Personality and Social Psychology	Peer Review	Discussion of collective effervescence as perceived emotional synchrony and four empirical studies, including one longitudinal and one experimental in process and outcomes of CG and CB
Páez et al. https://www.doi.org/10.1174/021347413804756078	2013	International Journal of Social Psychology	Peer Review	Social Identity and Collective Emotions in the framework of 15M.

Research problem

How to increase participation in collective behaviour and increase its positive effects on personal and social well-being?

Research objectives

- To measure the level of participation in collective behaviour, the processes involved in it and its short (CB), medium and long-term (SM) effects.
- To examine the factors that lead to participation in SM and its effects on personal and social well-being.

This text presents the variables and instruments that can be used to analyze the processes that occur in collective encounters (CG) and behaviour (CB), such as demonstrations, collective rituals, meetings, celebrations linked or not to social movements (SM)

To examine the level of participation and the factors that lead to participation in SM and its effects on personal and social well-being, a second set of instruments is presented. See figure 1

Figure 1 presents the "push variables" or psychosocial factors of participation in SM, conducive to participation in a serie of specific CB. Effect sizes values are based on the integration of meta-analysis. There are no values for positive emotions and the effect size of collective motives is based on only 4 studies - this is a limited estimate.

The last box presents the process variables that explain the microsocial effects of collective meetings. These cause positive emotional effects and generation of collective emotions, social integration (collective identity reinforcement), empowerment (efficacy, self-esteem, well-being) and increase agreement with social beliefs and values. As can be seen, CB participation reinforces push factors - at least in a successful flow and mobilization cycle.

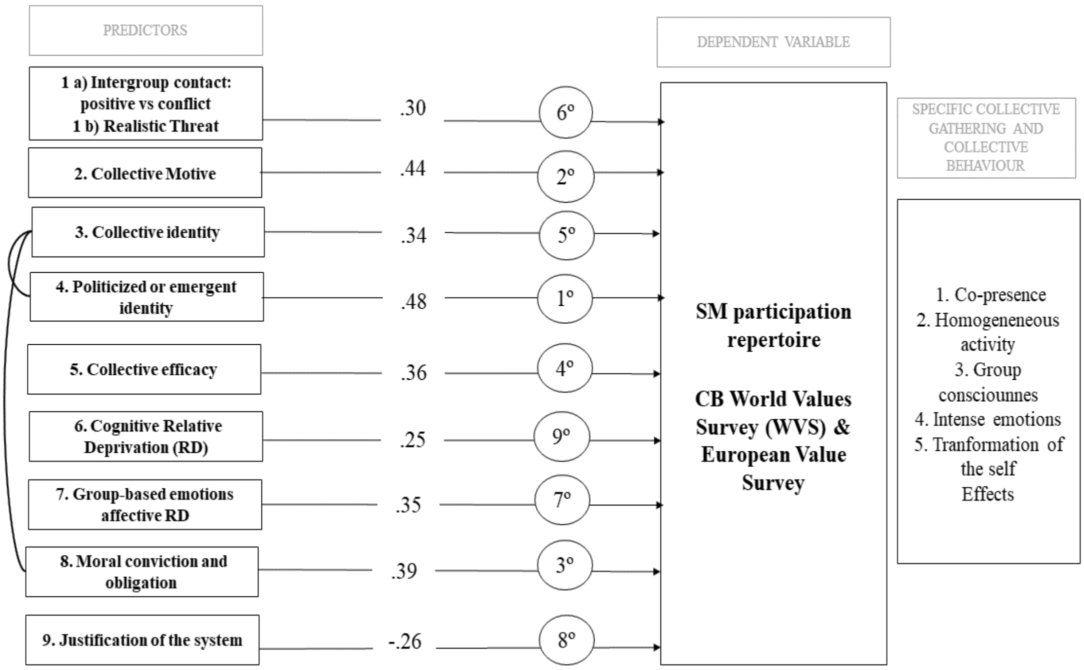

Figure 1. Psychosocial explanatory factors of SM, level of participation and processes during specific CG and CB

These are factors that motivate and facilitate participation(s) in a series of CB linked to an SM. In the scales it is necessary to define a reference group or SM (e.g. women and the feminist movement, students and the student movement, workers and the trade union movement...) As these must have a theme or SM to which reference is made, it has been illustrated with the case of the feminist movement, however, it should be adapted to the SM under study.

First, two repertory lists of CB conducted within the framework of SM are described. These are presented retrospectively, but can be used as behavioural intention or prospective.

Second, a cognitive free-association task is posed to collect indicators of social representations of SM.

Third, variables and instruments are described for factors that motivate and reinforce participation in SM: intergroup conflict / realistic threat, agreement with goals or collective motive, collective identity and efficacy (in a more general version of those used in CB), fraternal relative deprivation or perceived injustice of the in-group situation, politicized collective identification or with emerging mobilized groups, group-based

emotions (anger, hope, pride), beliefs of moral conviction and obligation, and ideological beliefs of system justification.

All of these factors are interrelated and positively associated with participation in contentious social change SMs - minus system justification beliefs, which are negatively associated with the above and positively associated with status-quo defense SMs. With the exception of the former instruments measuring level of participation in SM, the factors presented here predict regular participation in SM-linked CB. Higher participation, if the SM does not decline or brutally fail, in turn reinforces them.

Participation in SM can lead to improvements in emotions (a collective emotion variable and instrument is described), increased social integration and cohesion (measurable with social support and loneliness scales at the personal level and with social capital scales at the micro level), reinforcement of well-being, self-esteem and personal and collective self-efficacy (measurable with psychological and social well-being scales; personal and collective post-stress growth; self-efficacy and self-esteem scales as described) and ideological change or commitment (agreement with values, e.g., PVQ21, ideological positions, etc.). These scales are not presented but are available in the CCERG group.

A second set of instruments includes, first, a retrospective list of participation in collective and mass events. It allows for measuring the level of participation in CG and CB. Second, a guide to describe the CE that the person considers most relevant is described. Then, based on the overall experience assessed by the list, or based on the description of the most relevant CG, the sequence of processes and short-term effects occurring in CBs is answered by means of a set of short scales (between 3 and 6-9 items): situated social identity; perception level of shared attention; behavioural synchrony; perceived emotional synchrony (sense of unison and shared intense emotions); fusion of identity with group of the CG; intense positive emotions, emotions of transcendence of self (awe, elevation, moved by love), and negative ones, such as anger, stress and sadness (relevant in negative valence like mourning rituals or ambivalent like demonstrations CB); experience of transcendence and connection to values. Optimal experience or shared flow and the state of emotional novelty/creativity are also evaluated.

Finally, more general effects of social integration and empowerment are assessed, such as general identification with the in-group, collective efficacy and commitment to act in

favour of the group. As an example of a criterion variable, the PHI well-being scale (Hervás & Vazquez, 2013) is described and as a trait variable a short version of the ECI –S emotional creativity scale (Soroa et al; 2015). Other co-variables (such as PVQ21 values, Schwartz, 2007; spirituality Aspires (Simkin & Piedmont, 2018), Big Five personality traits (Ramsted & John, 2007) and attachment styles (Brennan et al., 1998) can be used as complements and according to the objectives of those applying the protocol. These variables and instruments allow for the measuring of the level of participation in CB, the processes involved in them and their short-term effects.

Some of these instruments -collective identity and collective efficacy- contain factors that facilitate continued participation in CB and, therefore, in SM. Generally, those who study SM are not interested in evaluating a specific type of collective behaviour, but rather in knowing the intensity and extent of participation in general collective behaviour linked to SM. With this in mind, the collective identity and collective efficacy instrument is often used; politicized collective identity; relative deprivation; group-based emotions; moral conviction and system-justifying or system-critical beliefs are also measured.

Methodology

PARTICIPATION IN SOCIAL MOVEMENT (SM)

SM participation repertoire (includes frequency and variety) (adapted from Klandermans, 1997; Castro-Abril et al., 2021).

The items are presented retrospectively. Prospectively, their formulation would be: "I am willing, within the framework of the feminist movement, to demonstrate, to send, to shout ..."

In relation to the SM "movement for the defense of women's rights", please answer if you have participated in the following ways.

1. I have demonstrated in public places peacefully.	1	2	3	4	5
2. I have demonstrated in public places and I have tried to defend myself against the actions of the forces of law and order.	1	2	3	4	5
3. I have sent messages, information and opinions through social networks.	1	2	3	4	5
4.I have shouted, chanted or played music from my house or building.	1	2	3	4	5

CB World Values Survey (WVS) & European Value Survey

WVS scale ask for the participation in CB in general last year. Alternative put a SM as a reference.

In relation to SM the "movement for the defense of women's rights" answer if you have participated in the following ways.

Forms of participation	Have Done (1)	Could Do (2)	Would never do it (3)
Have participated in the last year (or would be willing to)	1	2	3
1. Sign a petition	1	2	3
2. Participate in a boycott	1	2	3
3. Participate in a legal demonstration	1	2	3
4. Participate in a legal strike	1	2	3
5. Participate in an illegal strike	1	2	3
6. Occupy buildings or factories	1	2	3
7. Participating in illegal or unauthorized demonstrations in confrontation with the police.	1	2	3

<u>Psychosocial factors of SM</u>

Social representations measured with free word association (see Castro-Abril et al., 2021).

Free association of three words to stimuli linked to the SM. E.g. Feminists, Women, Demonstrators. Recorded and analyzed with lexical programs such as Iramuteq[3] or Alceste[4].

1A. Intergroup contact: positive vs conflict (Islam & Hewstone, 1993; Correa et al., 2021).

Higher negative perceived intergroup contact, associated to participation on SM. Higher positive perceived intergroup contact, associated to lower participation on SM for disavantadged groups. Opposite for high status groups (Cakal et al., 2011; Hassler et al., 2020). Exogroups are groups that share the opposite position from the participant regarding the controversial issue (e.g., if the participant is in favour of abortion, the out group was described as against abortion…), or share opposite/different ideological group, are from different social class, ethnic and national groups.

Ítems of the scale of contact with people from the group with opposing opinions or ideology, another social category (example used working class with upper class)

To what extent do you disagree or agree with the following statements in relation to the out group (upper social class)? Use the following scale to answer: Totally disagree to = 1 2 3 4 5 6 7=Strongly agree

[3] http://www.iramuteq.org/
[4] https://www.image-zafar.com/Logicieluk.html

1. I have felt close relationships with people of other social class	1	2	3	4	5	6	7
2. I have felt the relationships with people of other social class as competitive	1	2	3	4	5	6	7
3. I have felt the relationships with people of other social class as conflictive	1	2	3	4	5	6	7

1B. Realistic Threat out group Adaptation of Outgroup Realistic Threat Facet by Navas et al. (2012) from Stephan & Renfro (2002)

To what extent do you feel that, because of (anti-abortion groups), the following issues are in jeopardy? Use the following scale to answer: Totally disagree to = 1 2 3 4 5 6 7=Strongly agree

1. The economic situation of people like you	1	2	3	4	5	6	7
2. The health of people like you	1	2	3	4	5	6	7
3. The personal safety of people like you	1	2	3	4	5	6	7

2. Relevance attributed to the goal, objective or collective motive of a social mobilization

The collective motives, which are the value of the objective of the action (how much gender equality is valued, the right to abortion in the case of the feminist movement or independence in the case of Basque or Catalan nationalism, for example) and the expectation of success (about the number of participants and the probability of victory of the movement if many people participate). Subjects are asked about a) the value of the objective or collective good: importance given to a political or social objective and, b) the expectation of obtaining it. The collective motive is the multiplication of value by expectation.

<u>Value:</u>

How important is it to you? Use the following scale to answer

Not important at all =1 2 3 4 5 6 7=Very important

1. Gender equality	1	2	3	4	5	6	7
2. The right to abortion	1	2	3	4	5	6	7

<u>Expectations</u>

1. If many people participate, the Government and Parliament will be forced to agree to the changes<br>Totally disagree to = 1 2 3 4 5 6 7=Strongly agree	1	2	3	4	5	6	7
2. How many people do you expect to participate?<br>Few = 1 2 3 5 6 6 7=A lot or many	1	2	3	4	5	6	7

Collective motive is obtained by multiplying the two evaluation items of the objective by the expectation of participation and impact.

3.Collective Identity (see in the section on collective behaviours, Leach et al., 2008)

To what extent do you disagree or agree with the following statements in relation to the general group with which you carried out the collective activity/s? (Women in this example)

Responses were given on a seven-point scale ranging from: strongly disagree = 1 2 3 4 5 6 7 = strongly agree.

To what extent do you agree/disagree with the following statements.

1.I feel a bond with (...) women.	1	2	3	4	5	6	7
2.I feel solidarity with (...) women	1	2	3	4	5	6	7
3.I feel a (moral) commitment to (...the women)	1	2	3	4	5	6	7

4. Politicized or emergent identity (Agostini & van Zomeren, 2021; Páez & da Costa, 2022).

Responses are given on a seven-point scale ranging from: strongly disagree = 1 2 3 3 4 5 6 7 = strongly agree. To what extent do you agree/disagree with the following statements.

1. You identify with the people (students, women, Basques...) who participate in the feminist (student, nationalist...) movement.	1	2	3	4	5	6	7
2. You feel that you have a bond with the people (students, women, Basques...) who are involved in the feminist movement.	1	2	3	4	5	6	7
3. You see yourself as part of the feminist movement (student, nationalist...).	1	2	3	4	5	6	7

5. **Collective efficacy** (see in the section on collective behaviour).

To what extent do you disagree or agree with the following statements in relation to the general group with which you carried out the collective activity/s?

1. I believe that together we can change the current situation.	1	2	3	4	5	6	7
2. I believe that we are capable of achieving our goals.	1	2	3	4	5	6	7
3. I believe we can fight for our rights successfully.	1	2	3	4	5	6	7
4. I believe that we can influence political decisions.	1	2	3	4	5	6	7

6. **Relative Deprivation**. Relative Deprivation Scale RD (Obaidi et al., 2018).

Responses are given on a seven-point scale ranging from:

strongly disagree = 1 2 3 3 4 5 6 7 = strongly agree.

To what extent do you agree/disagree with the following statements.

1. Women (people of color, Muslims, the poor...) should have the same opportunities to improve their lives as men (white people, non-Muslims, the rich...).	1	2	3	4	5	6	7
2. Women (Muslims...) will always be at the bottom of the social ladder and men (non-Muslims...) at the top.	1	2	3	4	5	6	7
3. I am angry about the limited opportunities for women (Muslims...) to get ahead in life.	1	2	3	4	5	6	7
4. I think women (Muslims...) are disadvantaged because men (Western countries...) oppress them.	1	2	3	4	5	6	7
5. The women (Muslims...) are disadvantaged because the men (Western countries...) dominate them.	1	2	3	4	5	6	7
6. I am angry that (non-Muslim...) men discriminate against (Muslim...) women.	1	2	3	4	5	6	7

7. **Group-based emotions of anger, hope and pride.**

Emotions of anger-helplessness-hope scale (used in Zumeta et al., 2020).

In relation to recent events (e.g., on women's rights), to what extent do you feel the following emotions? Please use the following scale: not at all = 1 2 3 4 5 6 7 = to a great extent.

1. Do you feel indignation about the current situation (of women)?	1	2	3	4	5	6	7
2. Do you feel anger about the current situation (of women)?	1	2	3	4	5	6	7
3. Do you feel helpless about the current situation (of women)?*	1	2	3	4	5	6	7
4. Do you have hope that collective actions in favor of (women) will change the situation?	1	2	3	4	5	6	7
5. Do you feel pride in collective actions in favor of (women)?	1	2	3	4	5	6	7
Note. *to reverse							

8. **Moral conviction and obligation** (Sabucedo et al., 2018; used in Zumeta et al 2020).

Cause of mobilization must be defined.

To what extent do you disagree or agree with the following statements in relation to the topic or SM of reference...? Please use the following scale: not at all = 1 2 3 4 5 6 7 = totally agree

<u>Moral conviction of the participant</u>

1. My opinion about [violence against women...] is important to me.	1	2	3	4	5	6	7
2. I believe that my opinion about [...] is an important part of my moral standards and values.	1	2	3	4	5	6	7
3.I believe that my opinion about [...] has a moral character.	1	2	3	4	5	6	7
4. My opinion about [...] reflects an important part of who I am.	1	2	3	4	5	6	7

<u>Moral obligation</u>

1. To mobilize against (violence against women) is a moral obligation to oneself.	1	2	3	4	5	6	7
2. Regardless of what others think, I feel a moral obligation to participate in mobilizations (against violence against women).	1	2	3	4	5	6	7
3. Mobilizing against [...] would make me feel proud of myself.	1	2	3	4	5	6	7
4. I feel morally obliged to mobilize [...] even if it means confronting people close to me.	1	2	3	4	5	6	7

9. **System Justification** (Jost et al., 2003 in Vargas-Salfate et al., 2018).

To what extent do you disagree or agree with the following statements regarding the society in which you live. Please use the following scale: completely disagree = 1 2 3 3 4 5 6 7 = completely agree.

1. In general, society seems fair to me	1	2	3	4	5	6	7
2. In general, the political system in my country works as it should.	1	2	3	4	5	6	7
3. Everyone in my country has a fair chance at wealth and happiness	1	2	3	4	5	6	7
4.Society in my country is set up in such a way that people usually get what they deserve	1	2	3	4	5	6	7

Effects or Correlates

Effects of SMs can be evaluated with criteria variables relevant to them or with general scales such as the CEPN emotional climate scale (Páez et al., 1997).

Please, evaluate the state of your country, using the following scale: 1 = not at all 2 = little 3 = regular 4 = quite a lot 5 = very much

1.The economic situation in my country is very good.	1	2	3	4	5
2.The general emotional climate or environment in my country is very good.	1	2	3	4	5
The social climate or environment is of:					
3. Hope, hopeful	1	2	3	4	5
4. Solidarity, mutual help.	1	2	3	4	5
5. Trust in institutions	1	2	3	4	5
6. Fear, avoidance, worry.	1	2	3	4	5
7. Anger, hostility, aggressiveness among people	1	2	3	4	5
8. Sadness, passivity, low moods	1	2	3	4	5
9. Feel joyful, trust, contentment	1	2	3	4	5
10.Feel freedom to speak	1	2	3	4	5

COLLECTIVE BEHAVIOUR (CB)

Frequency of collective encounters (adhoc) (see a version in Cusi et al., 2022).

How often do you attend/ participate /attend collective meetings, mass events or social ceremonies? Please answer thinking about the last year

Use the following response range 0 = never 1 = sometime a year 2 = sometime a month 3 = once a week 4 = more than once a week

Large event or group meeting	0	1	2	3	4
1. I attend family or friends meetings or dinners with a large number of people.	0	1	2	3	4
2. I meet with people to go to concerts, musicals, movies, the theater or the like.	0	1	2	3	4
3. I attend union meetings	0	1	2	3	4
4. I participate in neighborhood or community meetings.	0	1	2	3	4

5. I participate in party meetings	0	1	2	3	4
6. I participate in association meetings	0	1	2	3	4
7. I attend social gatherings that involve religious practices, such as going to church, praying in a group, studying religious scriptures, etc. on a regular basis (e.g., weekly, monthly).	0	1	2	3	4
8. I attend particular events that occur very sporadically and involve religious practices, such as pilgrimages (Lourdes, Mecca) or a special mass ceremony with the Pope, etc.	0	1	2	3	4
9. I participate in parades or festivities, canivals and the like, attending, as a spectator, an artistic festival (e.g., music, art, gastronomy, etc.) that involves more than one day.	0	1	2	3	4
10. I attend rallies or protest demonstrations, e.g. political, street demonstrations due to a particular event.	0	1	2	3	4
11. I attend political rallies organized by a political party or movement; commemorations of different characteristics.	0	1	2	3	4
12. I attend, as a spectator, a sporting event, such as a soccer match, rugby, basketball, tennis, etc.	0	1	2	3	4
13. I attend leisure activities in a group (playing sports, going for a walk, dancing, etc.).	0	1	2	3	4
14. I participate in leisure activities such as meetings to discuss books, play board games, etc., or in activities to share common experiences.	0	1	2	3	4
15. I attend group meetings - periodic or occasional - aimed at helping myself or another person, such as group therapy, self-help groups, Alcoholics Anonymous meetings, or other group meetings.	0	1	2	3	4

Description of an important collective activity (adhoc)

Read very carefully the following definition of a collective meeting:

Collective meetings or gatherings are described as instances in which we participate collectively with a common goal and being aware of the other people participating in the activity. This goal may be for family or friends entertainment (e.g., collective festive lunch), religious (e.g., going to church, group prayer), political interest (e.g., attending a political meeting or rally), entertainment (e.g., attending a concert or soccer game), or because it is a necessity (e.g., attending weekly Alcoholics Anonymous meetings, or group therapy sessions). Although they are very different, during these collective activities, we are fully aware of the people who participate and, sometimes, we can live very intense and meaningful experiences.

<u>Examples of collective events</u>

1. Below are some relevant events by country and/or region, type of event, approximate date of the event and link to the event. We invite you to continue to add to this list of examples.
2. More frequent events such as family celebrations, formal and informal meetings of all kinds, are not specified. For example, "meeting of CCOO (Trade Union in

Spain) or CGT in France (trade Union in France)"[5] is not mentioned. CB associated with SM such as May 1, March 8[6] or the national or patriotic holidays of each country do not appear in this table either. These types of collective encounters, although relevant, are also common and general and therefore can also be evaluated/measured.

[5] Unión meetings

[6] International Workers' Day and International Women's Day

Event	Country/region	Type of event	Approximate date	Event Link
**Dressage and Folklore Festival “Jesús María”**	Cordoba/Argentina	Dressage and Folklore Festival	January	https://www.festival.org.ar/ ; others in this country https://www.cuandopasa.com/
**“Tamborrada/ Drumming”**	Donostia/San Sebastian/Basque Country/Spain	City's main celebration	January, 20th	https://danborrada.donostiakultura.eus/es/
**Carnival**	Tenerife, Spain	Festivity of International Touristic Interest	January/February	https://carnavaldetenerife.com/
**Carnival**	Uruguay	Popular celebration	January/February/ March	https://montevideo.gub.uy/carnaval-y-llamadas https://www.inclusion.gob.es/cartaespana/es/noticias/Noticia_0132.htm
**Festival “Cosquín Rock” (CRaño)**	Cordoba/Argentina It is also carried out in other countries such as Chile	Music Festival	February	https://festivalvillamaria.com/festivales-y-fiestas/cosquin-rock ; https://cosquinrock.net/
**“Tricota cultural”**	Córdoba/Argentina	Artistic meeting with strong social and cultural content	February	https://soundcloud.com/user-638536127/13-tricota-cultural
**Carnival**	Badajoz, Spain	Festivity of International Touristic Interest<br>More identity and popular festival	February/March	https://www.carnavalbadajoz.es/
**Rock in Rio**	Brazil and Portugal	Originally from Brazil, it includes rock and pop concerts. Known worldwide as "The world's largest festival"		https://rockinrio.com/rio/pt-br/home/; https://www.festicket.com/es/festivals/rock-in-rio/
**Carnival**	Brazil		February/March	https://home.centraldocarnaval.com.br/; https://www.riocarnaval.org/pt/
**“International Women's Day”**	International	Human rights	March, 8th	https://hacialahuelgafeminista.org/
**“Lollapalooza”**	General: different parts of the world	Rock Concert	2023: March in Argentina, Brazil and Chile; August in Chicago	https://www.lollapaloozaar.com/ ; https://www.lollapalooza.com/; https://www.lollapaloozade.com/
**"Isabel de Segura's Weddings"**	Teruel, Spain		February/March	http://www.bodasdeisabel.com/w3/bodas/index_bodas.aspx

**April Fair**	Seville, Spain	"Expression of color and joy of the city."	April	https://www.sevilla.org/fiestas-de-la-ciudad/feria-de-sevilla
**Korrika**	Basque Country, Spain	The aim of the race is to raise awareness of the Basque language and to raise funds for its teaching in AEK's learning centres.	It is held every two years in March/April	https://www.aek.eus/es-es/blog-1/content/korrika
**"Pilgrimage of the Virgen del Rocío"**	Aragón/Huelva, Spain		May	https://virgendelrocio.net/romeria/
**Carnival**	Murcia, Spain	Festivity of International Touristic Interest	May/June/Julio/August	https://www.carnavaldeaguilas.org/
**Carnival**	Cadiz, Spain	Festival of International Tourist Interest	June 2 to 12	https://www.cadizturismo.com/eventos/carnaval-de-cadiz
**"International Gay Pride Day"**	Madrid, Spain	Events that different collectives carry out publicly, to fight for the equality and dignity of gays, lesbians, bisexuals and transsexuals	June, 28th	https://www.diainternacionalde.com/ficha/dia-orgullo-gay
**A summer of festivals**	Spain		Summer	https://www.spain.info/es/top/fiestas-verano-espana/
**"The Angel's Heifer"**	Teruel, Spain		July	https://www.xn--interpeasteruel-4qb.es/index.php/la-vaquilla
**Aste Nagusia fest**	Basque Country/ Spain	Main festivals of the city	August	https://haycosasmuynuestras.com/semana-grande-pais-vasco/
**"Diego's departure"**	Teruel, Spain	National holiday of national tourist interest	September/October	http://www.bodasdeisabel.com/W3/Partida/Index_Partida.aspx
**"Carnival of blacks and whites"**	Southwestern Colombia	Popular festival Intangible Cultural Heritage of Humanity	December 28 at January 6	https://ich.unesco.org/es/RL/el-carnaval-de-negros-y-blancos-00287
**Folklore Rambles**	Belgium			
Others local, national, international				
Note: in "shaded" events that have been measured or are being measured				

Full description of an event

After reading this definition, we ask you to try to remember a specific moment in which you have lived it and, above all, one of which you remember as many details as possible. Which of the events in the list does your example match? Please mark an X where applicable

1.	I attend family or friends meetings or dinners with a large number of people.
2.	I meet with people to go to concerts, musicals, movies, the theater or the like.
3.	I attend union meetings
4.	I participate in neighborhood or community meetings.
5.	I participate in party meetings
6.	I participate in association meetings
7.	I attend social gatherings that involve religious practices, such as going to church, praying in a group, studying religious scriptures, etc. on a regular basis (e.g., weekly, monthly).
8.	I attend particular events that occur very sporadically and involve religious practices, such as pilgrimages (Lourdes, Mecca) or a special mass ceremony with the Pope, etc.
9.	I participate in parades or festivities, carnivals and the like, attending, as a spectator, an artistic festival (e.g., music, art, gastronomy, etc.) that involves more than one day.
10.	I attend rallies or protest demonstrations, e.g. political, street demonstrations due to a particular event.
11.	I attend political rallies organized by a political party or movement; commemorations of different characteristics.
12.	I attend, as a spectator, a sporting event, such as a soccer match, rugby, basketball, tennis, etc.
13.	I attend leisure activities in a group (playing sports, going for a walk, dancing, etc.).
14.	I participate in leisure activities such as meetings to discuss books, play board games, etc., or in activities to share common experiences.
15.	I attend group meetings - periodic or occasional - aimed at helping myself or another person, such as group therapy, self-help groups, Alcoholics Anonymous meetings, or other group meetings.
16.	Other
Specify:	

The protocol may have issues common to the whole society (workers, civilians, sportsmen, students, armed forces personnel and security forces personnel) and others specific to the sample being studied (for example, security or defense personnel), that is, although it can be applied to all types of sample, the results will surely be different, so it should be adapted.

For example, civilians in general do not participate in parades or ceremonies as usual practices (although there are folkloric or cultural parades, such as the gay pride parade). Similarly, members of the security forces and armed forces do not participate in partisan acts, protests, or political rallies - although in some countries they are unionized).

Write the date you attended the event.

Date [ ] Duration in hours [ ] Number of participants [ ]

In the space below, please describe that instance or event as specifically as possible.

- Why was this event relevant to you?

- What did the activity consist of?

- What happened during the activity?

- How did you participate?

- Was it something you usually do or was it the first time you did it?

- How did you feel during the activity?

- Was there a "peak" moment, like a "climax" during the period you were in that activity?

The order in which the instruments are presented below follows the sequence of the collective encounters according to Durkheim's model developed by Rimé & Páez (2023)

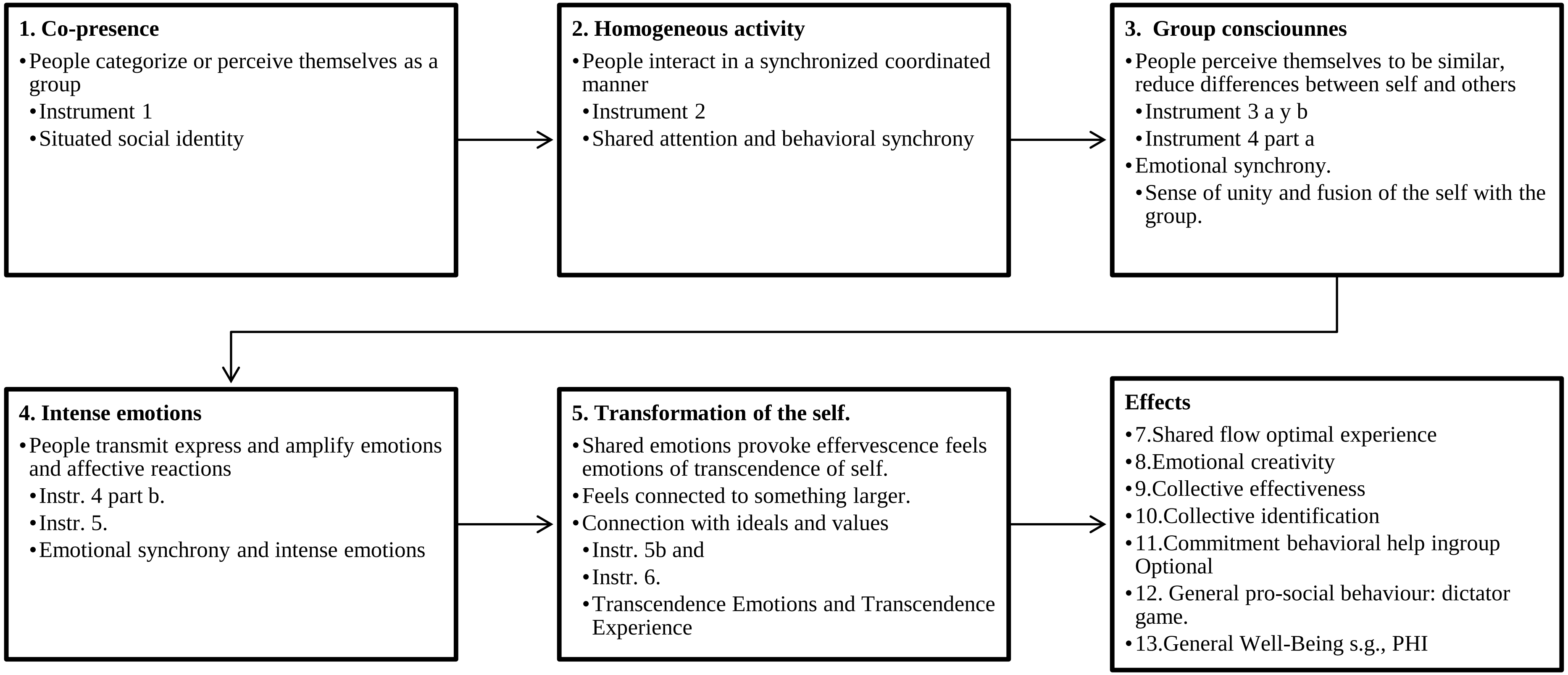
1. Co-presence
People categorize or perceive themselves as a group
Instrument 1
Situated social identity
2. Homogeneous activity
People interact in a synchronized coordinated manner
Instrument 2
Shared attention and behavioral synchrony
3. Group consciounnes
People perceive themselves to be similar, reduce differences between self and others
Instrument 3 a y b
Instrument 4 part a
Emotional synchrony.
Sense of unity and fusion of the self with the group.
4. Intense emotions
People transmit express and amplify emotions and affective reactions
Instr. 4 part b.
Instr. 5.
Emotional synchrony and intense emotions
5. Transformation of the self.
Shared emotions provoke effervescence feels emotions of transcendence of self.
Feels connected to something larger.
Connection with ideals and values
Instr. 5b and
Instr. 6.
Transcendence Emotions and Transcendence Experience
Effects
7.Shared flow optimal experience
8.Emotional creativity
9.Collective effectiveness
10.Collective identification
11.Commitment behavioral help ingroup Optional
12. General pro-social behaviour: dictator game.
13.General Well-Being s.g., PHI

Scale of explanatory processes and effects of meetings or collective encounters and rituals

This is the version about a specific activity

Answer based on the experience you (have) described/described.

Indicate how often you have had the following experience(s) during the group or collective activity (ceremony, demonstration, celebration, ritual or public meeting).

I. CO-PRESENCE SELF-CATEGORIZATION

1. Identity or Situated Social Identification (Identification with the participants of the collective meeting, Novelli et al., 2013).

Participants were asked about their degree of identification with the demonstrators. Response ranges ranged from: strongly disagree = 1 2 3 4 5 6 7 = strongly agree. The reliability coefficient score was very high in a nine-nation study of the 8M (Zumeta et al., 2020).

1. I identified with the other members of the crowd who were at the group activity.	1	2	3	4	5	6	7
2. I am like the other people who were in the collective activity.	1	2	3	4	5	6	7
3. I felt strong bonds with the other people who were in the collective activity.	1	2	3	4	5	6	7

II. HOMOGENEOUS ACTIVITY

2. Attentional and behavioral antecedents of synchronization in meetings

Shared attention and behavioral synchrony in encounter (based on Collins, 2014; Rennung & Göritz, 2016; Gabriel et al., 2017; Wlodarczyk et al., 2020 - Extended PES scale): 7 items (shared attention (3) and behavioral synchrony (4).

A Likert scale was used, with a response range from "not at all= 1 2 3 4 5 6 7 = very much"

The reliability coefficient was adequate in a nine-nation study of 8M demonstrations (Zumeta et al., 2020).

The people who participated in the collective gathering:							
1.Attended to the same symbols, objects or events	1	2	3	4	5	6	7
2.Simultaneously concentrated or focused their attention on the same symbols, objects or events	1	2	3	4	5	6	7
3.Attended to certain aspects of the event at the same time.	1	2	3	4	5	6	7
4.Carried out a coordinated activity	1	2	3	4	5	6	7
5.They did the same thing at the same time, such as clapping, dancing, laughing, praying, cheering, etc	1	2	3	4	5	6	7
6.They carried out a synchronous or convergent activity in time	1	2	3	4	5	6	7
7.They were acting in harmony							

III. REDUCTION OF DIFFERENCES WITH OTHERS/ SIMILARITY

3. Fusion of identity with the participants in the collective encounter: pictorial (Swann et al., 2009 in Gómez et al., 2011; 2012) and verbal (Gómez et al., 2011). Reliability was satisfactory in a study on the 8-M (Zumeta et al., 2020).

3.a.Verbal identity fusion scale.

During the collective or group activity, to what extent did you have the following thoughts using the following scale strongly disagree = 1 2 3 4 5 6 7 = strongly agree

My group is me	1	2	3	4	5	6	7
My group and I are one	1	2	3	4	5	6	7
I feel immersed in my group.	1	2	3	4	5	6	7
I feel a strong emotional bond with my group.	1	2	3	4	5	6	7
I make my group stronger	1	2	3	4	5	6	7
I would like to do more for my group than any other member of my group would like to do.	1	2	3	4	5	6	7

3.b.Pictorial identity fusion scale (Swann et al., 2009; 2012).

To assess identity fusion, the Pictorial Identity Fusion Scale (Gomez et al., 2011) was used, based on the measure "Inclusion of the Other in the Self (IOS) Scale" (Aron et al., 1992), this consisted of a pictorial item showing the perception of closeness or fusion with a reference group. In the case of 8-M, three items were included "Which image best describes your relationship with: 1) each reference group (feminists) , 2) situated in the context (participants in the specific 8-M demonstration), and 3) as a general category (e.g., all women in the world...). The five response options range from A to E, where A symbolizes less perceived closeness or fusion (i.e., non-overlapping circles) and E is greater closeness or fusion (i.e., completely overlapping circles).

Which picture best describes how you see yourself and your group during the collective or group activity?

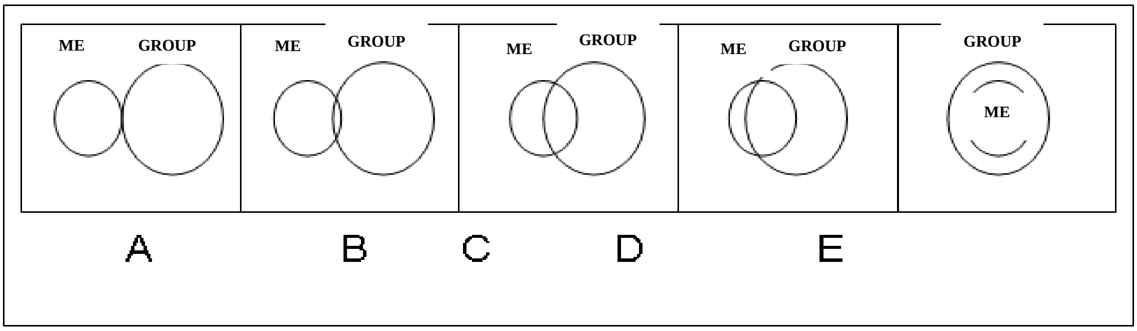

4. Perceived emotional synchrony in the encounter (Wlodarczyk et al., 2020).

A reduced six-item version (see Wlodarczyk et al., 2020) of the PES scale (Páez et al., 2015) was used to assess the perception of emotional synchrony with co-participants: sense of union (e.g. We have all acted as one person.) and intense sharing of emotion (e.g. We felt a strong shared emotion.). Cronbach's coefficient was high in the study by Zumeta et al., 2020.

4.a. Integration of individual self with the collective self is measured with PES sense of union 1, 2 and 3

Indicate how often you had the following experience(s) during the group or collective activity(ies) in which you participated (ceremony, demonstration, celebration, ritual or public encounter), taking into account that not at all= 1 2 3 4 5 6 7 = Very much

1. We have all acted as one person.	1	2	3	4	5	6	7
2. I felt that we were one person.	1	2	3	4	5	6	7
3.We felt that we were a whole.	1	2	3	4	5	6	7
4.We have felt more sensitive to the emotions and feelings of other people who feel the same way.	1	2	3	4	5	6	7
5.We felt a strong shared emotion.	1	2	3	4	5	6	7
6.We have felt stronger emotions than we usually do.	1	2	3	4	5	6	7

IV. TRANSMISSION, EXPRESSION AND AMPLIFICATION OF EMOTIONS

4.b. Is measured with the PES shared emotional intensity facet (items 4, 5 and 6).

5.a Intense positive emotions felt in the encounter (Novelli et al., 2013; adapted in Wlodarzyck et al., 2020). Three positive emotion items from the Novelli et al. (2013) scale referring to feeling fulfilled, happy and alive during the collective encounter were used. The response range ranged from: not at all = 1 2 3 3 4 5 6 7 = totally. Cronbach's reliability coefficient was high in previous studies (Castro-Abril et al., 2021).

At the time of the collective activity/s, I felt

1. Fulfilled	1	2	3	4	5	6	7
2.Happy.	1	2	3	4	5	6	7
3.Alive, active.	1	2	3	4	5	6	7

5.b. Transcendence and negative emotions felt in the encounter (DES scale, Fredrickson, 2009 based on Izard, 1982).

Fredrickson's (2009) Positivity Test was used to assess the positive emotions of transcendence through 4 items (5, 6, 7 and 9) and anger, stress and sadness (10 to 12). An item from Zickfeld's Kama Muta (4) and another from the CEPN scale (8) were added. This resulted in the brief scale of emotions of self transcendence (Fredrickson, 2009, adapted in Wlodarzyck et al., 2020). The response range ranged from: not at all = 1 2 3 3 4 5 6 7 = totally

At the time of the collective activity/s of to what extent have you felt the following feelings?

1.Moved, touched	1	2	3	4	5	6	7
2.Awe, wonder, amazement in something grand an vast	1	2	3	4	5	6	7
3.Morally inspired, uplifted or elevation	1	2	3	4	5	6	7
4.Love, closeness, trust	1	2	3	4	5	6	7
5.Solidarity, supporting others	1	2	3	4	5	6	7
6.Hopeful, optimistic, encouraged	1	2	3	4	5	6	7
7.Angry, irritated or annoyed	1	2	3	4	5	6	7
8.Stressed, nervous or overwhelmed	1	2	3	4	5	6	7
9.Sad, downhearted or unhappy.	1	2	3	4	5	6	7

V. TRANSFORMATIVE EXPERIENCE

6. Transcendent experience, connection to values, beliefs and ideals

Four items were used to measure the degree of transcendence experienced by people during a demonstration or collective encounter (Gabriel et al., 2020, adapted by Wlodarzyck et al; 2020). The reliability coefficient was very satisfactory in a nine-nation study on 8 M (Zumeta et al., 2020).

Indicate how often you have had the following experience(s) during the group or collective activity(ies) in which you participated (ceremony, demonstration, celebration,

ritual or public meeting), taking into account that strongly disagree = 1 2 3 4 5 6 7 = strongly agree

1. I felt that the event had an important purpose or objective.	1	2	3	4	5	6	7
2. I felt that there was something associated with values and ideals in the event.	1	2	3	4	5	6	7
3. I felt there was something special about the event.	1	2	3	4	5	6	7
4. I felt as if the event had changed me in some way.	1	2	3	4	5	6	7

Effects: Complex Behavioural Processes

7. Shared flow. It occurs in those SM and CB that potentially involve a challenge. The scale, which can be seen in full in Zumetan et al. (2022), was reduced to 6 items based on a psychometric analysis. The facets of the flow that are measured by an item on the scale are underlined in these protocol

Antecedents: manageable challenge, merging awareness action and clear goals

Processes: feedback, concentration, control

Psychological effects: changes in self awareness, time and positive experience

Indicate how often you have had the following thoughts or feelings during the group activity(ies), taking into account that: never = 1 2 3 4 5 6 7 = always

1. We were sure of what we wanted to do.	1	2	3	4	5	6	7
2. We were totally focused on what we were doing.	1	2	3	4	5	6	7
3. We felt that we could control what we were doing.	1	2	3	4	5	6	7
4. We all found the experience we had together very valuable and comforting.	1	2	3	4	5	6	7
5. We felt that we were good enough to meet the challenge or difficulty of the situation.	1	2	3	4	5	6	7
6. We were confident that, at the time, we were doing very well.	1	2	3	4	5	6	7

Effects: Complex emotional processes

8.Emotional creativity (ECI-S, Soroa et al., 2015) 9 items (3 from the novelty dimension (1, 2, 3 and 4), 3 from the emotional readiness dimension (1, 2, 6), 3 and 3 from the effectiveness-authenticity dimension (7, 8, 9). Readiness and authenticity act as trait and dispositional variables.

Three items of the ECI-S scale (Soroa et al., 2015) were used to assess the experience of creativity and emotional complexity during the collective encounter. Reliability was very

satisfactory in a study on protests in Chile (Castro-Abril et al., 2021) and other studies (da Costa, 2018).

Indicate how often you have had the following experience(s) during group or collective activity(ies) (ceremony, demonstration, celebration, ritual, or public encounter), taking into account that: 1 = very strongly disagree 2 = strongly disagree 3 = disagree 4 = agree 5 = strongly agree 6 = very strongly agree

1.I have felt a combination of emotions that other people have probably never experienced.	1	2	3	4	5	6
2.I have been able to experience a large number of different emotions.	1	2	3	4	5	6
3.I have had emotional experiences that could be considered unusual or out of the ordinary.	1	2	3	4	5	6

Regarding my emotions...						
4. When I have strong emotional reactions, I search for their reasons	1	2	3	4	5	6
5. I think about my emotional reactions and try to understand them	1	2	3	4	5	6
6. After an intensely emotional experience, I try to step back and examine my reactions objectively	1	2	3	4	5	6
7. My emotions are an important source of meaning in my life; without them, my life would be meaningless.	1	2	3	4	5	6
8. My outward emotional reactions I externalize adequately reflect my innermost feelings.	1	2	3	4	5	6
9. I am good at expressing my emotions.	1	2	3	4	5	6

Effects: Medium-term empowerment effects

9. Collective identity or long-term identification with the in-group (Leach et al., 2008). Leach's scale (multicomponent Group Identification) is composed of two major dimensions: self-definition (group homogeneity, individual self-stereotyping) and self-investment (centrality to identity, group satisfaction and group solidarity). It is this last subdimension (group solidarity) that has been used and is presented in this paper. Cronbach's alpha coefficients were very hight in the pre of a collective meeting (Tamborrada Donostia/San Sebastián) and in the post event (Páez et al., 2015).

Responses were given on a seven-point scale ranging from: strongly disagree = 1 2 3 4 5 6 7 = strongly agree.

To what extent do you agree/disagree with the following statements.

1.I feel a bond with (...) women.	1	2	3	4	5	6	7
2.I feel solidarity with (...) women	1	2	3	4	5	6	7
3.I feel a (moral) commitment to (...the women)	1	2	3	4	5	6	7

10.Collective efficacy (van Zomeren et al., 2010).

Four items taken from van Zomeren et al. (2010) were used in relation to the perception of the efficacy of the reference group, in this case, women (e.g., I believe that, together with women and men, we can change the current situation). The response range goes from: strongly disagree = 1 2 3 4 5 6 7 = strongly agree. Cronbach's alpha reliability coefficient was high in a nine-nation study of the 8 M (Zumeta et al., 2020).

To what extent do you disagree or agree with the following statements in relation to the general group with which you conducted the group activity/s:

1.I believe that together we can change the current situation.	1	2	3	4	5	6	7
2.I believe that we are capable of achieving our goals.	1	2	3	4	5	6	7
3.I believe we can fight for our rights successfully.	1	2	3	4	5	6	7
4.I believe that we can influence political decisions.	1	2	3	4	5	6	7

Effects: Long-term commitment to the in-group

11. Behavioral intention to help the group (Zumeta et al., 2020).

Five items were created to assess the behavioural intention of the participants to proactively collaborate in actions, organizations and initiatives in favour of the rights of the reference group. The response range is: 1 = not at all 2 = not very much 3 = regularly 4 = quite a lot 5 = a lot. The reliability coefficient was high in this study.

To what degree would you be willing to commit yourself to the following activities in favor of your collective, movement, group with which you carried out the collective activity(ies)

1.Participate in future collective actions or protests	1	2	3	4	5
2.Provide your email address to be invited to future collective actions	1	2	3	4	5
3.Commit 2 hours a week to collaborate with an association that organizes mobilizations.	1	2	3	4	5
4.Wear an emblem, symbol (badge, clothing etc.)	1	2	3	4	5

If you are willing to participate, you can give your email (write): ____________________________

12.General pro-social behaviour: dictator game.

To assess prosocial behaviour in general, an economic game is used, in which the degree to which money is shared is the indicator. In the dictator game (DG), the first player, "the dictator," determines how to divide an endowment (such as a cash prize) between themselves and the second player (the recipient).

The player decides the crew, which ranges from giving nothing to giving the entire crew. The recipient has no influence on the outcome of the game, which means that the recipient plays a passive role.

Sometimes the addressee is an NGO and other times it is a person. For instance Yudkin et al. (2022)

assessed prosocial behaviour using dictator game by providing participants with 10 tickets that would be redeemable for valuable prizes. Participants in secular mass gathering decided how many tickets they wanted to give to an anonymous stranger. Average donation was 62% of the tickets, a level that is notably higher than donations typically observed in dictator games, which average around 28%. Higher transcendent experience or transformative experience during secular ritual was associated to higher donation in DG post-test and sixth months following (Yudkin et al., 2022).

Eventually, a lottery or raffle could be held among the people who participate in this survey to assign a $100 bonus…if you won it, how much of that money would you keep for yourself and how much would you donate to a humanitarian NGO like MSF Doctors without frontiers or similar?

Amount of money for me	
Amount of money for a humanitarian NGO	

Effects: Well-being.

Keyes (social well-being), emotional climate CEPN (Páez et al., 1997 (short) or CD-24 by de Rivera, 2002 (long) and Phi (Pemberton Happiness Index by Hervás & Vázquez, 2013) for individual well-being. The latter scale is shown here.

Please say/say to what extent do you agree/s agree with the following statements. Use the following scale: strongly disagree = 0 1 2 3 4 5 6 7 8 9 10 = strongly agree.

1. I feel very satisfied with my life.	0	1	2	3	4	5	6	7	8	9	10
2. I feel that I have the energy to perform my daily tasks well.	0	1	2	3	4	5	6	7	8	9	10
3. I feel that my life is useful and valuable.	0	1	2	3	4	5	6	7	8	9	10
4. I feel satisfied with the way I am.	0	1	2	3	4	5	6	7	8	9	10
5. My life is full of learning and challenges that make me grow.	0	1	2	3	4	5	6	7	8	9	10
6. I feel very close to the people around me.	0	1	2	3	4	5	6	7	8	9	10
7. I feel capable of solving most of the problems of my day to day life.	0	1	2	3	4	5	6	7	8	9	10
8. I feel that I can be myself in the most important things.	0	1	2	3	4	5	6	7	8	9	10
9. I enjoy many little things every day.	0	1	2	3	4	5	6	7	8	9	10

10. In my day to day life I have many times when I feel bad.	0	1	2	3	4	5	6	7	8	9	10
11. I feel that I live in a society that allows me to develop myself fully.	0	1	2	3	4	5	6	7	8	9	10

Issues to consider in future research.

Measuring sex and cross-cutting gender, race, disability. Sex and gender have been used interchangeably in some of the research and sometimes, when gender is mentioned, sex is measured, and to a lesser extent the reverse is true (see Stock, 2022). The socio-demographic data generally includes a scale of ideological position from left 1 to right 10. This question cannot be applied to a sample of military or police personnel for legal reasons.

To facilitate replicability: observe construct content, response scale and label.

The response range of the different scales could pose a difficulty when coding the variables, so it is suggested that a transformation of the scale be applied (see page 200 in Páez et al., 2011[7]). We would especially appreciate it if any type of change made to the instrument could be clearly specified in the publications, as well as a description of means, SD, reliability and intercorrelations.

Administrative aspects

Once published, the protocol is freely accessible, however, we ask you to cite the published article. If you are interested, please contact the responsible researcher whose contact details are given below. We also ask that if you carry out research using the protocol, please let us know about your experience and results. Thank you very much

[7] In order to compare instruments with the same or similar items, but with different response scales, it is useful to transform the scores into a decimal scale (values between 0 and 10) and/or centesimal scale (from 0 to 100). To do this, the following algorithm is used: [Direct Score obtained minus Minimum Scale Score] divided by the Possible Range of the Scale] multiplied by [10 (for the decimal scale) or by 100 (for the centesimal scale). In other words, the minimum scale score is subtracted from the direct score. The possible range or minimum score minus the maximum score is calculated. And this is multiplied by ten or one hundred. Let us take an example in which two researchers have used the GHQ-12, but one of them has rated the responses with scores ranging from 1 to 5, and the other with scores ranging from 1 to 7. In the first case, the possible range of the sum of scores would range from 12 to 60, while in the second case it would range from 12 to 84. Let's consider that in the first response format a person obtained a direct score (PD) of 16. The decimal score in the first case would be obtained as follows: direct score minus minimum (16-12) = 4 divided by the range (60-12) of 48 = 0.083. Multiplied by 10 = 0.83. In the second case: direct score minus minimum (18-12) = 16 divided by the range (84-12) of 72 = 0.22. Multiplied by 10 = 2.22. That is, through this type of transformation we can find a homogeneous measurement scale with which to compare results from different studies using different ranges.

Continent	Country	Autonomous region or city	Responsible contacts	Variables of study or interest
**Europe**	Spain	Basque Country: Bilbao, Donostia/SS, Victoria/Gazteiz	dario.paez@ehu.eus	SM y CB adults
		Aragón: Teruel	sdacosta@unizar.es	SM y CB adults/ youth/organisations/security forces
		Catalunya: Barcelona, Girona	xavier.oriol@udg.edu	CB – adults/youth
	France		p.bouchat@uclouvain.be	CB adults
	Polond		anna.wlodarczyk@ucn.cl	CB adults
	Portugal		catarina.nslcarvalho@gmail.com	CB adults
	UK and India		h.cakal@keele.ac.uk	SM y CB adults
**Latinoamérica**	Argentina	Bs As	elenazubieta@hotmail.com	SM y CB adults
		Córdoba	marialorenalonso@gmail.com	CB adults, security forces
		Bs As	aletorres40@yahoo.com.ar	CB military field
	Brazil		elzamt400@gmail.com	CB
	Colombia		lopezlopezwilson@gmail.com	SM y CB
	Chile	Antofagasta	anna.wlodarczyk@ucn.cl	CB
		Santiago	mariajosemera@yahoo.es	CB
	Mejico	Guadalajara	nadhielii.alfaro@academicos.udg.mx	CB
	Peru		rcueto@pucp.pe	CB
	Uruguay		sdacosta@unizar.es	SM y CB adults/ youth/organisations/security forces

Cultura, Cognición y Emoción Reseach Group[8] and external collaborators

Ordered alphabetically by given name

Researcher	Institution	Project role	Other collaborations
**Alejandro Torres**	UNDEF, Argentian	Responsible for the military field	Co-responsible for the Americas and Latin America in the military field
**Alicia Izquierdo**	Full professor at University of Zaragoza, Teruel, Spain	Collaborator Spain, Aragon	
**Anna Wlodarczyk**	PDI CUN, Chile	Responsible for Chile and Poland	Expert assessor on Europe and Latin America
**Bernard Rimé**	Emeritus Professor, Catholic University of Lovain, Belgium	Expert assessor on Europe	Expert assessor on French and English languages and on Spain and Latin America
**Cakal Houseyin**	Full Professor University of Keele, UK	Responsible for UK and India	Collaborator on Spain (English)
**Camilo Unigarro**	Professor at University of La Sabana, Chía, Cundinamarca, Colombia	Collaborator Colombia	
**Catarina Carvalho**	Postdoctoral researcher, University of Oporto, Portugal	Responsible for Portugal	Collaborator on Spain and Latin America (Brazil)
**Darío Páez**	Professor at UPV/EHU, Donostia/San Sebastián, Spain	Responsible for Spain.	Expert assessor on Europe and Latin America
**Gisela Delfino**	Professor at UCB and Spain	Collaborator Spain and Argentina	
**Elena Zubieta**	Professor at UBA, Buenos Aires, Argentina	Co-responsible, Argentina, Buenos Aires-Conourbano	Collaborator Latin America and Europe (Spanish, English and French)
**Elza Techio**	Senior Lecturer University of Salvador de Bahía	Responsible for Brazil	Collaborator on Portugal and Latin America
**Ginés Navarro**	Full professor at University UJA, Spain	Co-responsible Andalucía, Spain	
**José J. Pizarro**	Researcher ACBC, Donostia/San Sebastian, Spain; Postdoctoral USAL, Spain	Collaborator ACBC Spain, co-responsible for Chile	Collaborator on Belgium and Latin America
**Lander Méndez**	Margarita Salas Postdoctoral Researcher, UV/EHU-UCN	Collaborator Spain-Chile	Collaborator on Portugal and Latin America
**Larraitz Zumeta**	Researcher ACBC, Urola Costa, Spain	Expert collaborator ACBC, Spain	Expert collaborator on Portugal and Latin America
**Laura Alfaro Beracochea**	Faculty, University of Guadalajara, Mexico	Responsible for Mexico	Collaborator on Spain
**Lorena Alonso**	Senior lecturer, National University of Villa	Responsible for Argentina	Collaborator on Spain

[8] https://www.ehu.eus/es/web/psicologiasocialcce

	Maria, Córdoba, Argentina		
**María José Mera (Coté)**	Full Professor Universidad Diego Portales, Chile	Co-responsible Chile	Collaborator Spain
**Marcela Murattori**	Full Professor UNDEF, Argentina	Co-responsible for military and civilian field, Argentina	Collaborator Spain civilian and military field
**María Rosario Cueto**	Senior lecturer, PCUP, Perú	Responsible for Peru	Collaborator on Spain
**MariCarla Martí González**	Postdoctoral researcher, Spain	Collaborator Spain (Zaragoza-Cantabria)	Collaborator Latin America (Cuba)
**Nekane Basabe**	Researcher ACBC, Bilbao, Spain	Expert assessor Spain	Expert assessor Europe (France) & Latin America
**Olaia Cusi**	PhD candidate, ACBC, Spain	Collaborator ACBC Spain	Collaborator América (Mexico)
**Pablo Castro**	PhD candidate, UPV/EHU, Spain-Colombia	Collaborator ACBC Spain, responsable for Colombia	Collaborator Latin America (Colombia, Mejico)
**Patricia Oberti**	Full Professor, Faculty, UDELAR, Uruguay	Collaborator Uruguay (Montevideo)	
**Pierre Bouchat**	Full Professor, Faculty, University of Lorraine, Bélgica	Responsible for France & Belgium	Expert collaborator on French and English languages and on Spain and Latin America
**Silvia da Costa**	Full Professor, Faculty, University of Zaragoza, España	Co-responsible Spain, responsible Aragón (Spain), Uruguay. Civilian population and security forces (police, military...)	Collaborator Spanish-speaking Europe & Latin America
**Virginia Díaz**	Full Professor, Faculty, UPV/EHU, Vitoria/Durango, Spain	Project collaborator Spain	Collaborator on English language
**Victoria Herrero**	Pre-doctoral Researcher	Collaborator Spain (Aragón)	
**Xavier Oriol**	Postdoctoral Professor-Researcher University of Girona, Spain	Co-responsible expert Spain, responsible Catalunya (Spain)	Expert collaborator Europe (English) and Latin America (Chile, Uruguay, Mexico and Peru)
**Wilson López**	Senior Lecturer, Colombia	Responsible Colombia	Collaborator Spain and Latin América
Abbreviations: ACBC = Autonomous Community of the Basque Country; PCUP = Pontifical Catholic University of Peru; SS = San Sebastian; CUN = Catholic University of the North; UPV/EHU = University of the Basque Country/Euskal Herriko Universitatea			

Bibliography[9]

Brennan, K. A., Clark, C. L., & Shaver, P. R. (1998). Self-Report Measurement of Adult Attachment: An integrative Overview. In J. A. Simpson &W. S. Rholes (Eds.), *Attachment Theory and Close Relationships* (pp. 46–76). New York: Guilford

[9] References not listed in the bibliography of the article are added here.

Collins, R. (2004). Interaction Ritual Chains. Princeton University Press. Collins, R. (2009). *Cadenas de Rituales de Interacción*. Madrid: Anthropos.

Correa, P., Brussino, S. & Reina, C. (2021). Adaptación de una Escala para Evaluar Contacto entre Ciudadanos de Distinta Clase Social del Gran Córdoba (Argentina). *Psicología, Conocimiento y Sociedad, 11*(2), 33-56.

de Rivera, J. (1992). Emotional Climate: Social Structure and Emotional Dynamics. In K. T. Strongman (Ed.), *International Review of Studies on Emotion, 2,* 197–218. John Wiley & Sons.

Fredrickson, B. (2009). *Positivity*. New York, NY: Crown Publishers.

Gabriel, S., Naidu, E., Paravati, E., Morrison, C. D., and Gainey, K. (2020). Creating the Sacred from the Profane: Collective Effervescence and Everyday Activities. *J. Posit. Psychol. 15*, 129–154. https://www.doi.org/10.1080/17439760.2019.1689412

Gabriel, S., Valenti, J., Naragon-Gainey, K., and Young, A. F. (2017). The Psychological Importance of Collective Assembly: Development and Validation of the Tendency for Effervescent Assembly Measure (TEAM). *Psychol. Asses. 29*, 1349–1362. https://www.doi.org/10.1037/pas0000434

Gómez, A., Brooks, M. L., Buhrmester, M. D., Vázquez, A., Jetten, J., and Swann, W. B. Jr. (2011). On the Nature of Identity Fusion: Insights into the Constructand a New Measure. *J. Pers. Soc. Psychol. 100*, 918–933. https://www.doi.org/10.1037/a0022642

Hervás, G., & Vázquez, C. (2013). Construction and Validation of a Measure of Integrative Well-Being in Seven Languages: The Pemberton Happiness Index. *Health and Quality of Life Outcomes, 11*(1), 66. https://www.doi.org/10.1186/1477-7525-11-66

Islam, M. R., & Hewstone, M. (1993). Dimensions of Contact as Predictors of Intergroup Anxiety, Perceived Out-Group Variability, and Out-Group Attitude: An Integrative Model. *Personality and Social Psychology Bulletin, 19*(6), 700-710. https://doi.org/10.1177/0146167293196005

Izard, Caroll E. (1982). *Measuring Emotions in Infants and Children*. Cambridge: Press Syndicate of the University of Cambridge.

Leach, C. W., van Zomeren, M., Zebel, S., Vliek, M. L., Pennekamp, S. F., Doosje, B., et al. (2008). Group-level Self-definition and Self-investment: A Hierarchical(multicomponent) Model of In-Group Identification. *J. Pers. Soc. Psychol. 95*:144. https://www.doi.org/10.1037/0022-3514.95.1.144

Navas, M., Cuadrado, I. & López-Rodríguez, L. (2012) Fiabilidad y evidencias de validez de la Escala de Percepción de Amenaza Exogrupal (EPAE). *Psicothema 24*, 3, 477-482

Novelli, D., Drury, J., Reicher, S., & Stott, C. (2013). Crowdedness Mediates the Effect of Social Identification on Positive Emotion in a Crowd: A Survey of Two Crowd Events. *PLoS One 8*:78983. https://www.doi.org/10.1371/journal.pone.0078983

Obaidi, M., Bergh, R., Akrami, N., & Anjum, G. (2019). Group-Based Relative Deprivation Explains Endorsement of Extremism Among Western-Born Muslims. *Psychological Science, 30* (4), 596-605. https://www.doi.org/10.1177/0956797619834879

Páez, D., Javaloy, F., Wlodarczyk, A., Espelt, E., & Rimé, B. (2013). El Movimiento 15-M: Sus Acciones como Rituales, Compartir Social, Creencias, Valores y Emociones. [The 15-M Movement: its Actions as Rituals, Social Sharing, Beliefs, Values and Emotions.] *Revista de Psicología Social, 28*(1), 19-33. https://www.doi.org/10.1174/021347413804756078

Páez, D., Beristain, C.M., González, J.L., Basabe, N., & de Rivera, J. (2011). *Superando la Violencia Colectiva y Construyendo Cultura de Paz.* Madrid: Fundamentos.

Páez, D. et al (1997). Clima Emocional: Su Concepto y Medición Mediante una Investigación Transcultural. *Revista de Psicología Social, 12*(1), 79-98.

Ramsted, B. & John, O. P. (2007). Measuring Personality in One Minute or Less: A 10 item Short Version of the Big Five Inventory in English and German. *Journal of Research in Personality, 41*, 203-212.

Rennung, M., and Göritz, A. S. (2016). Prosocial Consequences of Interpersonal Synchrony: A Meta-analysis. *Z. Psychol. 224,* 168–189. https://www.doi.org/10.1027/2151-2604/a000252

Rimé, B. & Páez, D. (2023). Why We Gather: A New Look, Empirically Documented, at Émile Durkheim's Theory of Collective Assemblies and Collective Effervescence. *Perspectives on Psychological Science*, 1-25, forthcoming https://www.doi.org./10.1177/17456916221146388

Rimé, B., Yzerbyt, V., & Mahjoub, A. (2017). Perception of Emotional Climate in a Revolution: Test of a Multistage Theory of Revolution in the Tunisian Context. *Br J Soc Psychol. 56*(4):633-654. https://www.doi.org/10.1111/bjso.12204

Schwartz, S. H. (2007). Value Orientations: Measurement, Antecedents and Consequences Across Nations. In R. Jowell, C. Roberts, R. Fitzgerald, & G. Eva (Eds.), *Measuring Attitudes Cross-Nationally* (pp. 169–204). SAGE Publications. https://www.doi.org/10.4135/9781849209458

Simkin, H., & Piedmont, R. L. (2018). Adaptation and Validation of the Assessment of Spirituality and Religious Sentiments (ASPIRES) Scale Short Form into Spanish. *Latinoamerican Journal of Positive Psychology, 4*(1), 96-107.

Soroa, G., Gorostiaga, A., Aritzeta, A. & Balluerka, N. (2015). A Shortened Spanish Version of the Emotional Creativity Inventory (the ECI-S). Creativity Research Journal, 27(2), 232-239. https://www.doi.10.1080/10400419.2015.1030313

Stephan, W.G., & Renfro, C.L. (2002). The Role of Threat in Intergroup Relations. In D.M. Mackie and E.R. Smith (Eds.), *From Prejudice to Intergroup Emotions. Differentiated Reactions to Social Groups* (pp. 191-214). London: Taylor & Francis Group.

Stock, K. (2022). *Material Girls. Why Reality Matters for Feminis*. Barcelona: Shackleton Books.

Swann, W. B. Jr., Jetten, J., Gómez, Á, Whitehouse, H., & Bastian, B. (2012). When Group Membership Gets Personal: A Theory of Identity Fusion. *Psychological Review, 119*(3), 441-456.

Swann, W. B. Jr., Gómez, A., Seyle, C. D., Morales, J. F., & Huici, C. (2009). Identity Fusion: The Interplay of Personal and Social Identities in Extreme Group Behavior. *J. Pers. Soc. Psychol. 96*, 995–1011. https://www.doi.org/10.1037/a0013668

van Zomeren, M., Leach, C. W., & Spears, R. (2010). Does Group Efficacy Increase Group Identification? Resolving Their Paradoxical Relationship. *J. Exp. Soc. Psychol. 46*, 1055–1060. https://www.doi.org/10.1016/j.jesp.2010.05.006

Valencia, J. (1990). La Lógica de la Acción Colectiva: Tres Modelos de Análisis de la Participación Política no Institucional. *Revista de psicología social*, *5*(3), 185-214. [The Logic of Collective Action: Three Models of Analysis of Non-Institutional Political Participation

Vargas-Salfate, S., Páez, D., Khan, SS., Liu, J.H. & Gil de Zúñiga, H. (2018) System Justification Enhances Well-Being: A Longitudinal Analysis of the Palliative Function of System Justification in 18 Countries. *Br J Soc Psychol. 57*(3), 567-590. https://www.doi.org/10.1111/bjso.12254

Yudkin, D.A., Prosser, A.M.B., Heller, S.M., McRae, K., Chakroff, A. & Crockett, M.J. (2022). Prosocial Correlates of Transformative Experiences at Secular Multi-Day Mass Gatherings. *Nature Communications*, *13*, 2600. https://www.doi.org/10.1038/s41467-022-29600-1

Appendix 1

If you want to carry an essay or pilot study with this instrument, apply version (a) with the frequency of collective meetings. In case you want to measure a specific event, apply version (b) not including the frequency of collective meetings and focus questions in the specific CG.

Version a

- Frequency of collective encounters (adhoc).
- Description of an important collective activity (adhoc)
- Full description of an event
- Write the date you attended the event
- Answer based on the experience you (have) described/described.
- Indicate how often you have had the following experience(s) during the group or collective activity (ceremony, demonstration, celebration, ritual or public meeting) you described…

Version b

- Indicate how often you have had the following experience(s) during the group or collective activity (ceremony, demonstration, celebration, ritual or public gathering) in which you have participated.
