# Supplementary material for: Social movements and collective behavior: an integration of meta-analysis and systematic review of social psychology studies
Source: Front Psychol. 2023 Apr 21;14:1096877. doi: 10.3389/fpsyg.2023.1096877 (PMC10162496; doi:10.3389/fpsyg.2023.1096877)
Supplement: Supplementary file 3 [file Data_Sheet_3.PDF]

Complementary material 3

Selection of general reviews that were not integrated because they were not systematic or overlapped with the integrated reviews. For example, Rosa and Klanderman's article was a Portuguese version of a review of psychosociological theories of movements by Klandermans and collaborators in other texts, without adding a systematic review of Brazilian studies.

Aslanadis, P. (2012). Critical Review of Social Movement Literature. Available at. https://www.academia.edu/2381793/Critical_Review_of_Social_Movement_Literature; https://www.researchgate.net/publication/304489497_Critical_Review_of_Social_Movement_Literature

Fisher, D.R., Andrews, K.T., Caren, N., Chenoweth, E., Heaney, M.T., Leung, T., Perkins, L.N. & Pressman, J. (2019). The Science of Contemporary Street Protest: New Efforts in the United States. *Sci Adv.* *5*(10), eaaw5461. https://www.doi.org/10.1126/sciadv.aaw5461

Giugni, M., Bosi, L & Uba, K. (2020). *Outcomes of Social Movements and Protest Activities.* Oxford Bibliographies. https://www.doi.org/10.1093/OBO/9780199756223-0037 Retrieved from https://www.oxfordbibliographies.com/

Giugni, M. (2008). Political, Biographical, and Cultural Consequences of Social Movements. Sociology Compass. *Sociology Compass*, *2*(5), 1582-1600. https://doi.org/10.1111/j.1751-9020.2008.00152.x

Grauman, C.R. & Moscovici, S. (1986). *Changing Conceptions of Crowd Mind and Behavior*. New York: Springer Verlag.

Jaspers, J.M. (2014) Emotions, Sociology, and Protest. In Ch. von Scheve & M. Salmeda (Eds.). *Collective Emotions* (pp. 341-355). Oxford: Oxford Academic. https://www.doi.org/10.1093/acprof:oso/9780199659180.003.0023

Jaspers, J.M. (2017). The Doors That Culture Opened: Parallels Between Social Movement Studies and Social Psychology. *Group Processes & Intergroup Relations* *20*(3), 285– 302. https://www.doi/10.1177/1368430216686405

Jenkins, J.C. (1983) Resource Mobilization and the Study of Social Movements. *Annual Review of Sociology*, *9*:527-53

Manning, R.O. (1971) A Critical Analysis of Contemporary Collective Behavior Theory, *Sociological Focus*, *4*(4), 99-106. https://www.doi/10.1080/00380237.1972.10570796

Mariot, N. (2001). Les Formes Élémentaires de L'effervescence Collective, ou L'état D'esprit Prêté aux Foules. *Revue française de Science Politique*, 51, 707-738. https://www.doi.org/10.3917/rfsp.515.0707

Marx, G.T. (2012) Looking at Smelser's Theory of Collective Behavior After Almost 50 Years: A Review and Appreciation. *American Sociology, 43,* 135–152. https://www.doi.org/10.1007/s12108-012-9153-6

Passy, F. & Monsch, G.A. (2018). Biographical Consequences of Activism. In D. A. Snow, S.A. Soule, H. Kriesi & H.J. McCammon. *The Wiley Blackwell Companion to Social Movements* (Second Edition), (pp. 499-514). John Wiley & Sons Ltd.

Rosa, L.A. & Klandermans, B. (2022). Psicologia Social do Protesto: Um Panorama Teórico a partir da Realidade Brasileira. *Psicologia: Ciência e Profissão, 42,* e233201, 1-15. https://www.doi.org/10.1590/1982-3703003233201

Sawyer, J.E. & Gampa, A. (2022). Social Movements as Parsimonious Explanations for Implicit and Explicit Attitude Change. *Personality and Social Psychology Review* 1–24 https://www.doi.org/10.1177/10888683221095697

Snow, D.A., Vliegenthart, R. & Ketelaars, P. (2019). The Framing Perspective on Social Movements: Its Conceptual Roots and Architecture. In D.A. Snow., S.A. Soule., H., Kriesi & H.J. McCammon (Eds.). *The Wiley Blackwell Companion to Social Movements.* Second Edition. (pp. 392-410). New Yersey: Wiley Blackwell. https://www.doi.org/10.1002/9781119168577.ch22

Van Stekelenburg, J., Klandermans, B. and Walgrave, S. (2019). Individual Participation in Street Demonstrations. In D.A. Snow, S.A. Soule, H. K., and H.J.

McCammon. *The Wiley Blackwell Companion to Social Movements* (Second Edition) (pp. 371-391). John Wiley & Sons Ltd.

Vestergren, S. & Drury, J. (2022). Biographical Consequences of Environmental Activism. In M. Grasso & M. Giugni (Eds). *The Routledge Handbook of Environmental Movements*. London: Routledge. ISBN 9780367428785.
